# Chemokine-Adjuvanted Plasmid DNA Induces Homing of Antigen-Specific and Non–Antigen-Specific B and T Cells to the Intestinal and Genital Mucosae

**DOI:** 10.4049/jimmunol.1901184

**Published:** 2020-01-08

**Authors:** Yoann Aldon, Sven Kratochvil, Robin J. Shattock, Paul F. McKay

**Affiliations:** Department of Medicine, Imperial College London, London W2 1PG, United Kingdom

## Abstract

Plasmid DNA is a promising vaccine platform that together with electroporation can elicit significant systemic Ab responses; however, immunity at mucosal sites remains low. In this study, we sought to program T and B cells to home to the gastrointestinal and vaginal mucosae using genetic chemokine adjuvants and assessed their impact on immune homeostasis in various distinct immune compartments. BALB/c mice were immunized i.m. with plasmid DNA encoding a model Ag HIV-1 Env gp140 and selected chemokines/cytokine and boosted intravaginally with gp140 recombinant protein. Isolated splenocytes, intestinal lymphocytes, and genital lymphocytes as well as serum and intestinal luminal contents were assessed for Ag-specific reactivity. In addition, flow cytometric analysis was performed to determine the impact on immune homeostasis at these sites. Different molecular chemokine/cytokine adjuvants effected significant alterations to the recruitment of B and T cells to the spleen, vaginal and intestinal mucosae, for example CCL25 enhanced splenic and vaginal Ag-specific T cell responses whereas CCL28 increased the levels of specific T cells only in the vaginal mucosa. The levels of Ab could be modulated in the systemic circulation, as well as the vaginal vault and intestinal lumen, with CCL20 playing a central role. Our data demonstrate that the CCL20, CCL25, and CCL28 genetic chemokine adjuvants enhance the vaccine Ag-specific humoral and cellular responses and induce homing to the intestinal and female genital mucosae.

## Key Points

- Bicistronic Ag–molecular adjuvant vectors induce distinct immune profiles.
- CCL20 and CCL28 induce homing of Ag-specific T cells to the female genital tract.
- CCL20 and CCL25 induce homing of Ag-specific B cells to the intestinal mucosa.

## Introduction

The portals of entry for most pathogens are the mucosal surfaces, and in the case of HIV-1, the genital tract and the rectal mucosae are the primary sites of entry (1, 2). To date, efforts to develop efficient vaccines against HIV-1 have led to limited or no protection and even to an increased susceptibility to infection in human clinical trials (3). Whether it involves humoral and/or cellular responses, the ability to generate and maintain an appropriate immune response at mucosal sites is crucial to prevent infection (4–6). Although the majority of licensed vaccines are administered through the i.m. route and trigger high levels of systemic immunity, this delivery route is much less efficient at directing protective immune responses toward mucosal sites (7). Therefore, approaches to induce effective mucosal responses have generally focused on the use of chemokines and cytokines as molecular adjuvants for vaccines with the aim to recruit cells from the systemic compartment and program them for homing to mucosal surfaces (8–11).

Significant work has been put into stabilizing soluble HIV-1 Envelope (Env) immunogens to make them amenable to the clinic and large scale manufacturing (12–15). We and others have developed stabilized native-like trimers that can be delivered through DNA or RNA platforms (15–17) and immune responses induced by these trimers could be polished by recombinant protein boost immunization(s) at sites of interest. Nucleic acid-based, and in particular DNA-based, vaccines offer a great opportunity by limiting the extent of manufacturing and long-term storage considerations. Delivered on their own, DNA plasmids expressing particular Ags do not induce high levels of immunity, but electroporation (EP) along with genetic adjuvants have proven to be effective ways of inducing strong immune responses (18, 19).

In this study, we compared the potential of four chemokines and one cytokine at inducing homing of B and T cells to the intestinal and vaginal mucosae. These included CCL20/MIP-3α, which is secreted by mucosal epithelial cells and plays a key role in immune cell homeostasis (20); CCL25/TECK, which has been shown to induce homing of T cells and IgA Ab-secreting cells to the intestinal mucosa (21, 22); and CCL27/CTACK and CCL28/MEC, which are secreted by the skin and epithelial cells, respectively (23–25). These two chemokines bind to CCR10, which is present on the surface of T and B cells associated with mucosae, and along with CCL25, they proved to be effective molecular adjuvants in vaccine regimens that elicited protection against SIV in macaques (26). Finally, because of its broad proinflammatory and immune cell activation properties, we selected the TNF-α cytokine for evaluation in our study (27, 28).

With the idea of reducing manufacturing steps and multivector based vaccines, we produced a bicistronic expressing DNA vector using the internal ribosome entry site (IRES) of the encephalomyocarditis virus (EMCV), which allows coexpression of our Ag of interest along with a chemokine or cytokine. Using a model HIV-1 CN54gp140 soluble Env Ag, we evaluated the potential of the selected chemokines/cytokine to induce homing of Ag-specific immune cells to the intestinal and vaginal mucosae in a DNA prime-protein boost strategy. The DNA was administered i.m. with EP, and the protein boost was delivered to the female genital tract. We demonstrated in our present work that the designed bicistronic vector induces different immune responses depending on the molecular adjuvant encoded. Furthermore, we showed that our immunization strategy could induce homing of T cells to the vaginal mucosa (CCL20, CCL28) and B cells to the small intestine (CCL20, CCL25).

## Materials and Methods

### Vectors

Chemokine genes were created based on published sequences, synthesized (codon optimized for *Mus Musculus* expression), and cloned into pMK vector using GeneArt gene synthesis service (Thermo Fisher Scientific). The *CCL20–IRES–CN54gp140* and CN54*gp140–IRES–CCL20*-expressing vectors were designed in silico and cloned into pcDNA3.1(+) using GeneArt services. *CN54gp140* was derived from a clade C (CN54) HIV-1 Env isolate and expresses a soluble form of truncated Env glycoprotein. We positioned *CN54gp140* either 5′ of the IRES separated by a spacer, which includes three stop codons in the three reading frames after the cloning site followed by an intron, or 3′ of the IRES, where the RNA translation is under the control of the IRES (Supplemental Fig. 1A, 1B). The other CN54*gp140–IRES–chemokine* vectors were produced by cloning the chemokine/cytokine gene (*CCL25*, *CCL27*, *CCL28*, *TNF-α*, and *scrambled CCL20* [*scb*]) amplified by PCR with extended 5′ and 3′ ends to flank the gene with the appropriate restriction sites. Once digested with EcoRI-HF and XhoI-HF (New England Biolabs), the pcDNA3.1(+) *CN54gp140–IRES*–backbone and chemokine/cytokine gene inserts were run on a gel and purified using a gel purification kit followed by a PCR purification kit run (Qiagen). The *scb* gene was designed using the *CCL20* sequence where all ATG methionine codons were altered, the start codon being changed for a stop codon, and two additional stop codons were introduced in +2 and +3 reading frames to ensure that no translatable protein could be synthesized. Ligation was performed using the T4-ligase (New England Biolabs) and TOP10 chemically competent bacteria (Invitrogen). The plasmid DNA preparations were purified using the Plasmid Plus Maxi kit (Qiagen).

### Cell cultures and transient transfection

HEK 293T.17 cells (American Type Culture Collection) were maintained in complete medium using DMEM (Sigma-Aldrich) supplemented with 10% FBS, 2 mM glutamine, 100 U/ml penicillin G, and 100 μg/ml streptomycin (Life Technologies). Cells were handled in a sterile cabinet and cultured in a humidified, 5% CO_2_, +37°C incubator.

For transfections, HEK 293T.17 cells were seeded in T-75 flasks and transfected with 12 μg of plasmid DNA using PEI Max (Polysciences) with a 1:3 (w:w) DNA/PEI ratio. After 16–18 h, transfection medium was removed and FreeStyle 293 medium (Life Technologies) added (20 ml). Supernatants were harvested 48 h later and filtered (0.45 μm) after cellular debris were pelleted (5 min, 2000 × *g*). Filtered supernatants were stored at −20°C and then tested for gp140 and chemokine/cytokine expression by Western blot and ELISA, respectively.

### Animals and immunization procedure

BALB/c mice aged 6–8 wk old were placed into groups of *n* = 8. Animals were handled and procedures were performed in accordance with the terms of a project license granted under the UK Home Office Animals (Scientific Procedures) Act 1986. All the procedures and protocols used in this study were approved by an animal ethical committee, the Animal Welfare and Ethical Review Body. One animal in the *CN54gp140–IRES–-scb* group was euthanized at week 6 as it presented a tumor. Therefore, *n* = 7 animals for this group after week 6. In the *CN54gp140–IRES–CCL25* group, one animal was euthanized at week 9 because of a tumor, and thus *n* = 7 at the final time point (week 10.5) for this group.

Groups of mice were injected i.m. (quadriceps) with 10 μg of plasmid DNA in 50 μl PBS followed by EP using 5 mm electrodes using an ECM 830 Square Wave EP system (BTX) with three pulses of 100 V each, followed by three pulses of the opposite polarity with each pulse lasting 50 ms and an interpulse interval of 50 ms. Animals were immunized three times at 3-wk intervals and boosted intravaginally at week 9 with 10 μg CN54 gp140 protein + 10 μg monophosphoryl lipid A (MPLA). One week prior to the protein boost, mice were injected s.c. with hydroxyprogesterone (Makena) to prevent variability between animals with regard to their estrous cycle. Serum samples were collected at each immunization time point and 10 d after the last immunization. Mice were euthanized 10 d after the last immunization and organs processed.

### Organ processing and lymphocyte isolation

Ten days after the last immunization, mice were euthanized and organs were collected. Spleens were removed, placed into 5 ml RPMI 1640, and processed as previously described (29). Intestines were removed, and ∼10–15 cm were excised below the stomach from the duodenum down to the middle of the jejunum. The luminal content (LC) of this section of the intestine was collected by flushing 500 μl of 1× PBS through the segment into a 1.5-ml collection tube containing 15 μl of protease inhibitors (MilliporeSigma). The LC were then kept on ice during organ processing, followed by centrifugation at 5000 × *g* for 5 min to pellet feces before storage at −20°C until the ELISAs were performed. The isolation of lymphocytes from the small intestine was carried out as previously described (30). Briefly, Peyer's patches (PP) were removed with sharp scissors and place into 2 ml cold harvest medium. To isolate the intraepithelial lymphocytes (IEL) and lamina propria lymphocytes (LPL), the intestine was cut longitudinally and then into smaller transversal pieces (∼9 mm^2^) and placed into 20 ml of calcium magnesium–free buffer to wash off fecal matter. After several washes and digests with dithioerythritol, EDTA, and collagenase solutions at +37°C, cells were pelleted by centrifugation for 5 min at 400 × *g*. Cells were resuspended in 44% Percoll diluted in RPMI 1640 (Sigma-Aldrich) and then underlaid with 66.7% Percoll diluted in RPMI 1640 using a 16-gauge needle. Following centrifugation at 600 × *g* for 20 min at room temperature, lymphocytes were isolated from the 44 to 66.7% interface, rinsed, and pelleted for 5 min at 400 × *g*. The PP lymphocytes, IEL, and LPL of each animal were pooled and resuspended in 2 ml cold harvest medium, counted using trypan blue (Lonza), and were then ready for immunoassays. The vagina and upper genital tract up to the fallopian tubes were removed, placed into 5 ml of cold harvest medium, then cut into pieces and processed using the same method as for the IEL/LPL isolation. The cells were resuspended in 1 ml of complete medium, and 600 μl were used for the IFN-γ ELISpot, whereas the remaining 400 μl were used for the flow cytometry staining.

For B cell ELISpot, splenocytes were cultured for 72 h with R848 (1 μg/ml) (Invivogen) and IL-2 (10 ng/ml) (Roche) in 100 μl RPMI 1640 + 10% FBS at 2.5 × 10^5^ cells per well (two wells per sample).

### ELISpots

IFN-γ ELISpot. Assessment of the IFN-γ T cell response was performed using the Mouse IFN-γ ELISpot^PLUS^ kit (Mabtech) following the manufacturer’s instructions. Briefly, anti–IFN-γ precoated plates were blocked with complete medium for at least 30 min, then cells were added at 2.5 × 10^5^ cells per well for negative control (complete medium only) and CN54gp140 peptide pool (1 μg/ml) conditions in 200 μl final volume per well. The positive control wells contained 5 × 10^4^ cells per well in 200 μl final volume per well with 5 μg/ml of concanavalin A. Five times fewer cells were used for the positive control to obtain distinct spots following the polyclonal activation induced by concanavalin A. For the vaginal lymphocytes, 100 μl of the lymphocyte preparation was used per well, including for the control wells. The plates were incubated overnight at +37°C, 5% CO_2_, and developed as per the manufacturer’s protocol. Once dried, plates were read using the AID ELISpot reader ELR03 and AID ELISpot READER software (Autoimmun Diagnostika). Four spleen samples were damaged during processing (three in the CCL28 group and one in the TNF-α group) and were excluded from IFN-γ and memory B cell (mBC) ELISpot analyses. For the vaginal IFN-γ ELISpots, samples with <20 spot forming unit (SFU) in the positive control wells were excluded: one in the PBS group, one in the CCL27 group and two in the TNF-α group.

For the B cell ELISpot, ELISpot plates (MilliporeSigma) were activated with 15 μl/well 70% EtOH for 1 min. Following 5× 200 μl/well washes with sterile water, 5 μg/ml CN54gp140 protein diluted in 1× PBS in 100 μl/well was added for the Ag-specific wells, and 15 μg/ml of anti-mouse IgG (Southern Biotech) in 100 μl 1× PBS was added for the total IgG wells. The plates were incubated overnight at +4°C then washed 5× with 200 μl/well with 1× PBS and blocked with 200 μl/well of complete medium for 2 h at +4°C. R848 + IL-2 stimulated splenocytes were then added onto the plates. Each condition was tested in duplicate wells, and plates were incubated overnight for 16–18 h at +37°C, 5% CO_2_. After six washes with 200 μl/well 1× PBS, 100 μl/well of 1:5000 biotinylated goat anti-mouse IgG detection Ab (Southern Biotech) in 1× PBS + 0.5% FBS was added and incubated overnight at +4°C. The plates were then washed five times with 200 μl/well 1× PBS and 100 μl/well of 1:1000 streptavidin-HRP (Mabtech) in 1× PBS + 0.5% FBS added. Following a 1-h room temperature incubation, plates were washed again five times, and 100 μl/well of the AEC substrate (BD Biosciences) was added. The reaction was stopped with water after 10 min. Once dried, plates were read using the AID ELISpot reader ELR03 and AID ELISpot READER software (Autoimmun Diagnostika).

### Flow cytometry

For the splenocytes and intestinal lymphocytes, 1 × 10^6^ cells were used per staining panel for each animal. For the vaginal lymphocytes, 200 μl of the isolated lymphocyte population was used for each staining panel and each animal. Cells were stained with Aqua viability dye (1:400) (Molecular Probes) in FACS buffer (2.5% FBS, 1 mM EDTA, 25 mM HEPES in 1× PBS) for 20 min on ice, in the dark. The cells were then washed with FACS buffer, split into two tubes, pelleted at 600 × *g* for 5 min, and stained with panel 1 and 2 (Tables I, II) in 100 μl FACS buffer for 30 min on ice in the dark. Following two washes with FACS buffer, cells were resuspended in 100 μl 1× PBS and fixed with an additional 100 μl 3% paraformaldehyde (Polysciences), final 1.5%. A minimum of 15,000 and 25,000 gated live lymphocytes were acquired for samples from the spleen and intestine, respectively, and full samples were acquired for the vagina. The data were acquired on a LSR Fortessa flow cytometer (BD Biosciences) and analyzed using FlowJo v.10.1 (TreeStar) (Supplemental Figs. 3, 4A, 4B).

### SDS-PAGE Western blotting

Samples were prepared in Novex Tris-Glycine SDS sample buffer, boiled at +95°C for 5 min, and loaded onto a 4–12% polyacrylamide Tris-Glycine gels (Invitrogen). After transfer onto nitrocellulose membranes (Invitrogen), the membranes were blocked with blocking buffer (2% BSA in 1× PBS–0.05% Tween 20), then primary 2G12 human anti-Env IgG (1 μg/ml in blocking buffer) was added. Following three washes (1× PBS–0.05% Tween 20), a secondary goat anti-human IgG Fc biotinylated Ab was added (1:10,000 in blocking buffer) and incubated overnight at +4°C. The membranes were washed, incubated with streptavidin-ALP 1:1000 (Thermo Fisher Scientific) in blocking buffer and washed again before adding the BCIP/NBT substrate (Sigma-Aldrich). Reaction was stopped after 5–10 min with distilled water.

### Chemokine/cytokine ELISAs

Supernatant from HEK293T.17 cells transfected with chemokine/cytokine-expressing vectors were tested for the presence of different secreted chemokines/cytokine using the DuoSet ELISA kits following the manufacturer’s instructions (R&D Systems).

### Total and Ag-specific IgG, IgG1, IgG2a, and IgA ELISA

The Ag-specific IgG, IgG1, IgG2a, and IgA titers were assessed by ELISA as previously described (29). MaxiSorp high binding ELISA plates (Nunc) were coated with 100 μl/well of 1 μg/ml HIV-1 Env CN54gp140 (Polymun) in 1× PBS. For the standard IgG/IgG1/IgG2a/IgA, three columns on each plate were coated with 1:1000 dilution of goat anti-mouse κ and λ L chains (Southern Biotech). To measure total IgG and IgA, the plates were entirely coated with goat anti-mouse κ and λ L chains. After overnight incubation at +4°C, the plates were washed four times with 1× PBS–Tween 0.05% and blocked for 1 h at +37°C with 200 μl/well blocking buffer (1% BSA in 1× PBS–0.05% Tween 20). The plates were then washed, and the diluted samples or standard Igs (IgG, IgG1, IgG2a, or IgA) were added using 50 μl/well volume. The plates were incubated for 1 h at +37°C and washed four times, and secondary Ab was added at 1:2000 dilution in blocking buffer (100 μl/well) using anti-mouse IgG-HRP, anti-mouse IgG1-HRP, anti-mouse IgG2a-HRP, or anti-mouse IgA-HRP (Southern Biotech). After 1 h incubation followed by four washes, plates were developed using 50 μl/well TMB and the reaction was stopped after 5 min with 50 μl/well stop solution (Insight Biotechnologies). For intestinal luminal samples, quantification of total IgG and IgA content was assessed to normalize the Ag-specific IgG and IgA response to the total IgG and IgA content, respectively. The absorbance was read on a KC4 Spectrophotometer at 450 nm (BioTek Industries).

### Statistical analysis

Statistical analyses were carried out using a two-way ANOVA with Dunnett correction for multiple comparisons or Mann–Whitney unpaired *t* test to determine statistical significance using GraphPad Prism v7.0h.

## Results

### Characterization of the bicistronic expression vectors

To coexpress our Ag of interest along with chemokines, two bicistronic DNA expression plasmids were designed (Supplemental Fig. 1A, 1B). We used the EMCV IRES to produce DNA plasmids coding for two proteins from a single mRNA transcript. This bicistronic vector approach provides a clear advantage over the use of separate plasmids, as it ensures that any transfected cell, whether it remains at the injection site or is trafficked to the draining lymph node, will express both the Ag and the chemokine/cytokine. First, we tested whether the expression of the large HIV-1 Env CN54gp140 Ag (1959 bp) would be affected by its position upstream or downstream of the EMCV IRES along with the CCL20 chemokine expression. Analysis showed that expression of both proteins was optimal with the *CN54gp140* positioned upstream and *CCL20* downstream of the IRES (Ag–IRES–chemokine) as confirmed by Western blot and ELISA (Supplemental Fig. 1C, 1E). Therefore, we selected this configuration for our study and further cloned the genes coding for CCL25, CCL27, CCL28, TNF-α, and a *scb* control gene into the Ag–IRES–chemokine backbone. Expression of CN54gp140 and each chemokine/cytokine was confirmed and no chemokine could be detected from expression of the Ag–IRES–scb vector as expected (Supplemental Fig. 1D–F).

### Ag–IRES–chemokine plasmid DNA immunizations induce distinct humoral responses

**FIGURE 1.**
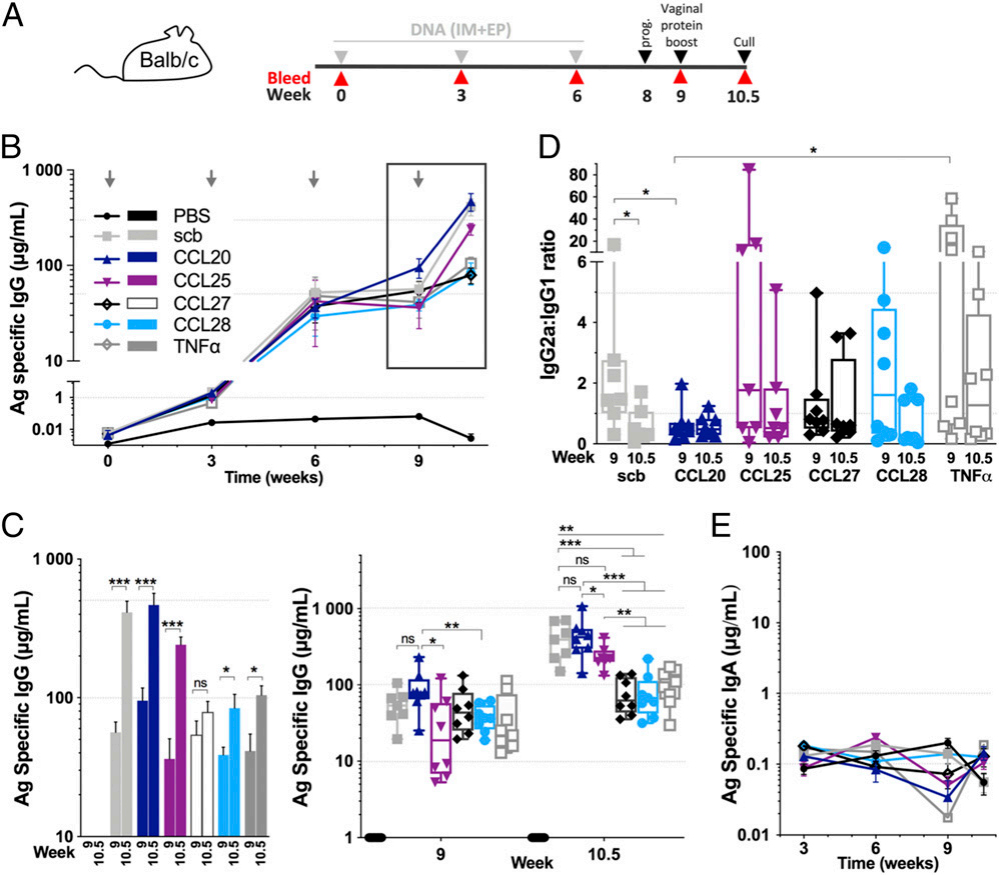
Systemic humoral responses induced by chemokine-adjuvanted DNA. (**A**) Schematic of the mouse study immunization schedule. Three DNA injections (gray arrows) 3 wk apart, followed by a s.c. injection of hydroxyprogesterone (prog.) 1 wk before the vaginal protein + MPLA boost (black arrowheads). Blood sample collection is indicated by red arrowheads. (**B**) CN54gp140 Ag-specific serum IgG ELISA. Mean ± SEM. (**C**) Serum ELISA results from week 9 after the three DNA injections and from week 10.5 for the final serum samples. The first panel shows statistical comparison between week 9 and 10.5 for each group (mean ± SEM), whereas the second panel shows statistical comparison between groups at this two time points (box and whiskers, min/max). (**D**) IgG2a/IgG1 ratio of the different groups for week 9 and week 10.5. Box and whiskers, min/max. (**E**) Quantification of the Ag-specific serum IgA response assessed by ELISA. Mean ± SEM, *n* = 8 animals per group and *n* = 7 from week 6 for Ag–IRES–scb and at week 10.5 for Ag–IRES–CCL25. Mann–Whitney *U* test. *p < 0.05, **p < 0.01, ***p < 0.001. ns, nonsignificant. See also Supplemental Fig. 2.

Immunogenicity studies were performed in BALB/c mice to evaluate the impact of chemokines/cytokine coexpression on the CN54gp140 Ag-specific cellular and humoral immunity. Groups of *n* = 8 animals were immunized three times, 3 wk apart, with 10 μg of plasmid DNA delivered i.m. + EP followed by an intravaginal protein boost at week 9. Serum samples were collected throughout the study, and mice were euthanized 10 d after the protein boost and organs processed for further analysis (Fig. 1A).

We first evaluated the Ag-specific IgG levels in the systemic compartment by ELISA. After two DNA injections, serum IgG levels were similar in all groups with titers of ∼20–50 μg/ml (Fig. 1B). After the third DNA injection, the Ag–IRES–CCL20 group reached the highest Ag-specific IgG level, which was significantly higher than Ag–IRES–CCL25 and Ag–IRES–CCL28 groups (Fig. 1C). The Ag–IRES–scb control group, which expresses CN54gp140 protein but no genetic adjuvant, induced good but lower levels of Ag-specific IgG compared with Ag–IRES–CCL20, although this was not statistically significant (*p* = 0.0939). Ten days after the intravaginal protein boost, the Ag–IRES–scb and –CCL20 groups showed high serum Ag-specific IgG, which were significantly higher than Ag–IRES–CCL27, –CCL28, and –TNF-α groups (*p* < 0.01). This difference was also significantly higher compared with Ag–IRES–CCL25 for the Ag–IRES–CCL20 group but not for the Ag–IRES–scb group. Interestingly, the Ag–IRES–CCL25 group, which had a similar IgG titer to Ag–IRES–CCL27, –CCL28, and –TNF-α groups at week 9, was efficiently and significantly boosted compared with these three groups. Among the genetically adjuvanted groups, the Ag–IRES–CCL25 induced the highest Ag-specific serum IgG boosting between week 9 and 10.5 with a ∼ 6.7-fold increase whereas Ag–IRES–CCL27, –CCL28, and –TNF-α were below 3 (Supplemental Fig. 2A). The substantial serum IgG titer enhancement observed for the Ag–IRES–CCL25 group suggests that after DNA immunizations immune cells might have homed to the vaginal mucosa where they could be activated following Ag exposure. These results suggest that *CCL20* adjuvanted DNA was able to induce a strong systemic humoral response that can be recalled/boosted upon Ag exposure at a distal mucosal site from the initial site of injection whereas *CCL27*, *CCL28*, and *TNF-α* genetic adjuvantation appeared to limit the induced systemic humoral response.

Next, we measured the IgG1 and IgG2a Ag-specific responses by ELISA to evaluate the type of Th response induced by our Ag–IRES–chemokine DNA immunizations (Supplemental Fig. 2B–D). The IgG2a/IgG1 ratio was used as a surrogate to gauge the Th1 and Th2 responses (Fig. 1D) (31). Following the three DNA injections, a slightly skewed response toward Th1 was observed for Ag–IRES–scb group whereas the Ag–IRES–CCL20 group showed a predominant Th2 response, which remained unchanged after the protein boost. Interestingly, Ag–IRES–scb switched to a more Th2-directed response after the intravaginal boost. In contrast to the Ag–IRES–CCL20 and –scb groups, the CCL25, CCL28, and TNF-α adjuvanted group induced a mixed Th1/Th2 profile by week 9, with about half of the animals in each group having either a Th1 or Th2 skew. This pattern was persistent for the Ag–IRES–TNF-α group after the protein boost, whereas Ag–IRES–CCL25 and –CCL28 groups showed a dramatic reduction in Th1 skewed responses. The CCL27 adjuvanted group maintained a more balanced Th1/Th2 profile following the protein boost. These results indicate that the molecular adjuvants tested can induce diverse Th1 or Th2 cellular responses. In addition, we demonstrate in this study that none of the groups were able to induce detectable Ag-specific IgA in the systemic compartment (Fig. 1E).

**FIGURE 2.**
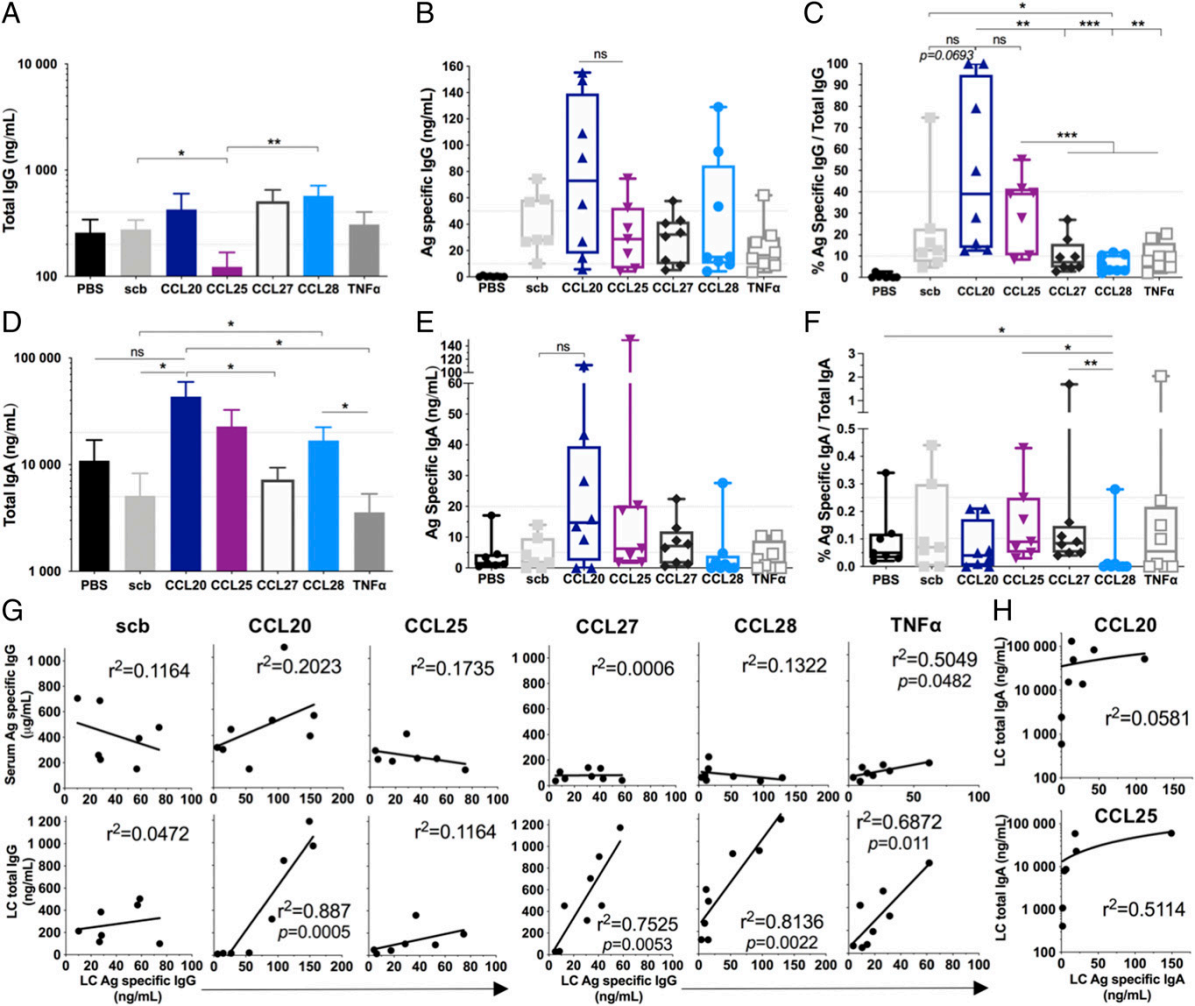
Intestinal humoral responses induced by chemokine-adjuvanted DNA. Quantification of the CN54gp140 Ag-specific and total IgG and IgA levels in the intestinal LC assessed by ELISA reported as (**A** and **D**) total IgG/IgA (nanogram per milliliter, mean ± SEM), (**B** and **E**) Ag-specific IgG/IgA (nanogram per milliliter, box and whiskers, min/max), and (**C** and **F**) percentage of total IgG/IgA (box and whiskers, min/max) with *n* = 8 animals per group and *n* = 7 for Ag–IRES–scb and Ag–IRES–CCL25. Mann–Whitney *U* test, *p < 0.05, **p < 0.01, ***p < 0.001. (**G**) Comparison of the serum and LC IgG Ag-specific responses (top row) and comparison of the LC total and Ag-specific IgG (bottom row) for Ag–IRES–scb, –CCL20, –CCL25, –CCL27, –CCL28, and –TNF-α groups. (**H**) Comparison of the LC total and Ag-specific IgA for Ag–IRES–CCL20 and –CCL25 groups. Black lines in (**G**) and (**H**) show the linear regression, with *r*^2^ values reported on each plot and *p* values indicated when statistically significant. ns, nonsignificant.

To further characterize the humoral response, we analyzed the Ag-specific IgG levels in the LC of the small intestine. We quantified the total and Ag-specific IgG by ELISA and normalized the specific response to the total IgG (Fig. 2A–C). Ag–IRES–CCL20, –CCL27, and –CCL28 groups presented higher levels of total IgG compared with the PBS and Ag–IRES–scb control groups, although not statistically significant (Fig. 2A). The Ag–IRES–CCL25 group showed the lowest total IgG level among all groups, and this was significant compared with Ag–IRES–scb and –CCL28 groups. When we looked at Ag-specific IgG in the LC, the CCL20 adjuvanted group presented the highest proportion of Ag-specific IgG, with four animals having ≥50% of their total IgG being specific for Env (Fig. 2C), and this was significantly higher (*p* < 0.01) than the CCL27, CCL28, and TNF-α adjuvanted groups (3–10% of specific IgG). The Ag–IRES–scb group showed good levels of LC Ag-specific IgG (Fig. 2B), with a median at 12.7% of the total IgG (Fig. 2C). The Ag–IRES–CCL25 group displayed a significantly higher percentage of specific IgG compared with Ag–IRES–CCL27, –CCL28, and –TNF-α groups, with a median at 39%. However, when we looked at the quantified specific IgG (Fig. 2B), there was no difference in quantified Ag-specific IgG between these groups. This indicated that although the Ag–IRES–CCL25 group had less total IgG in the LC, the produced IgG are proportionally more specific than for the Ag–IRES–CCL27, –CCL28, and –TNF-α groups. Importantly, linear regression analysis comparing the Ag-specific IgG levels in the serum and LC showed that there was no correlation between these two parameters except for the TNF-α group (Fig. 2G, top row). Moreover, there was no correlation between the total IgG and the Ag-specific IgG in the LC for the Ag–IRES–scb control group (*r*^2^ = 0.0472), whereas these two parameters were strongly correlated for Ag–IRES–CCL20 (*r*^2^ = 0.887, *p* = 0.0005), Ag–IRES–CCL27 (*r*^2^ = 0.7525, *p* = 0.0053), Ag–IRES–CCL28 (*r*^2^ = 0.8136, *p* = 0.0022), and Ag–IRES–TNF-α (*r*^2^ = 0.6872, *p* = 0.011) (Fig. 2G, bottom row). These data suggest that the chemokine-adjuvanted groups were able to program B cells to home to the intestinal mucosae but that this process was not solely restricted to the Ag-specific B cells.

Next, we characterized the IgA response in the LC. Interestingly, the Ag–IRES–CCL20 group presented significantly higher total IgA compared with Ag–IRES–scb, –CCL27, and –TNF-α groups (Fig. 2D). The Ag–IRES–CCL28 group also presented higher total IgA compared with Ag–IRES–scb and –TNF-α groups. Surprisingly, there was less Ag-specific IgA detected than specific IgG in all groups (Fig. 2B, 2E), with the CCL25 and CCL20 adjuvanted groups showing the highest levels of Ag-specific IgA. However, the Ag-specific IgA represented <0.5% of the total measured IgA (Fig. 2F). In addition, there was no correlation between the LC total and Ag-specific IgA for all groups (Fig. 2H, and data not shown). These data suggest that CCL20 and CCL25 genetic adjuvantation can induce homing of IgA secreting B cells to the intestinal mucosae, but again, this was not restricted to Ag-specific B cells.

### The systemic IFN-γ cellular response is reduced by CCL25 genetic adjuvantation

**FIGURE 3.**
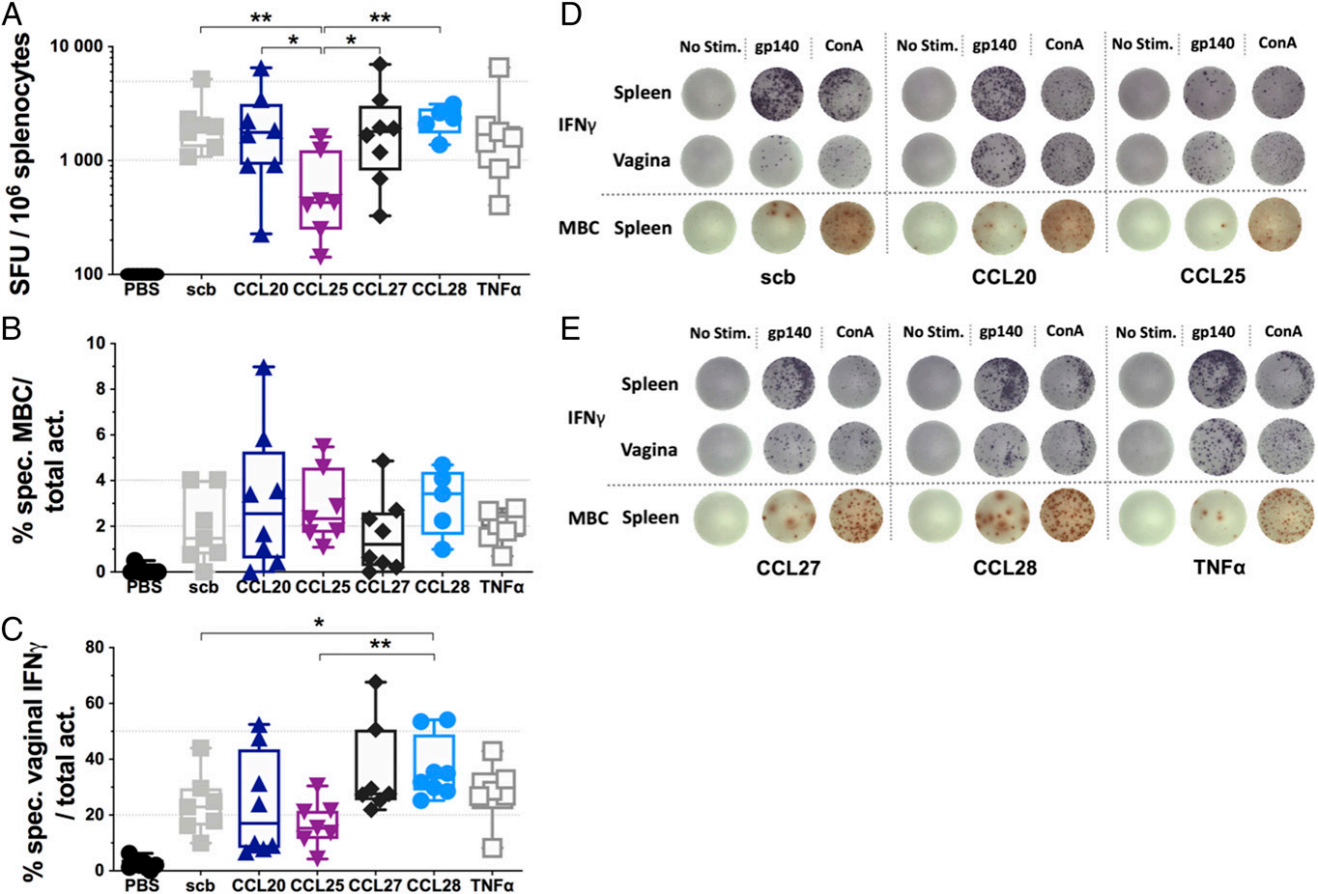
CCL25 limits the systemic cellular response whereas CCL28 promotes vaginal cellular responses. (**A**) Mouse IFN-γ ELISpot results of lymphocytes isolated from the spleen and stimulated with CN54gp140 peptide pool. (**B**) Mouse mBC ELISpot results using isolated lymphocytes from spleens. Spot counts are reported as percentage of specific mBC per total activated cells. (**C**) Mouse IFN-γ ELISpot results of lymphocytes isolated from the vaginal mucosa and stimulated with CN54gp140 peptide pool. (**D** and **E**) Representative images of the ELISpot wells for each group. Gp140 stands for gp140 peptide pool for the IFN-γ ELISpots and for the coated CN54gp140 protein for the mBC ELISpots. Box and whiskers, min/max. For (A and B), *n* = 8 for PBS, CCL20, and CCL27 groups; *n* = 7 for scb, CCL25, and TNF-α; *n* = 5 for CCL28. For (C), *n* = 8 for CCL20 and CCL28 groups; *n* = 7 for PBS, scb, CCL25, and CCL27; *n* = 6 for TNF-α (see *Materials and Methods*). Mann–Whitney *U* test. *p < 0.05, **p < 0.01. ConA, concanavalin A; No stim., no stimulation.

Subsequently, we performed IFN-γ ELISpots on splenocytes using CN54gp140 peptide pools to determine the influence of each chemokine/cytokine on the CTL/NK cell response. With a mean SFU of 646 per 10^6^ splenocytes, the Ag–IRES–CCL25 group led to a marked reduction (∼3.5-fold) in systemic Ag-specific killer cells compared with the Ag–IRES–scb group, whereas all the other genetically adjuvanted groups showed similar SFU counts compared with the Ag–IRES–scb group (Fig. 3A, 3D, 3E).

Then, we evaluated the mBC pool from the spleens using mBC ELISpots. Although there was no statistical difference between groups, the CCL20 adjuvanted group presented the highest mBC proportion, with ∼6 and 9% of the total mBC pool being specific for CN54gp140 in two of the animals (Fig. 3B). Of note, the CCL27 and CCL28 adjuvanted groups formed large spots indicating that the mBC clones were activated and produced large quantities of Abs whereas the Ag–IRES–CCL20 group formed clear small spots (Fig. 3D, 3E).

### CCL28 genetic adjuvantation promotes vaginal IFN-γ cellular responses

Following euthanasia, lymphocytes from the vaginal mucosa were isolated and IFN-γ ELISpots performed. Results showed that cells secreting IFN-γ upon Ag-specific activation represented a substantial proportion of the total lymphocyte population in the CCL28 adjuvanted group with a mean of ∼37% of the activated cells being Ag-specific (Fig. 3C, 3E). In contrast, the Ag–IRES–scb and –CCL25 group induced a significantly lower Ag-specific vaginal cellular response, with respectively ∼24% and ∼17% of the total activated cells being Ag specific. An increase in Ag-specific IFN-γ secreting cells was also noted for the Ag–IRES–CCL27 group but to a lower extent than the Ag–IRES–CCL28 group. Therefore, these results suggest that CCL28 genetic adjuvantation induced homing of Ag-specific CTL and/or NK cells to the vaginal mucosa whereas CCL25 adjuvantation seemed to limit homing of these cells to the vagina.

### CCL20 and CCL25 DNA-adjuvantation induce B cell homing to the intestinal mucosa

**FIGURE 4.**
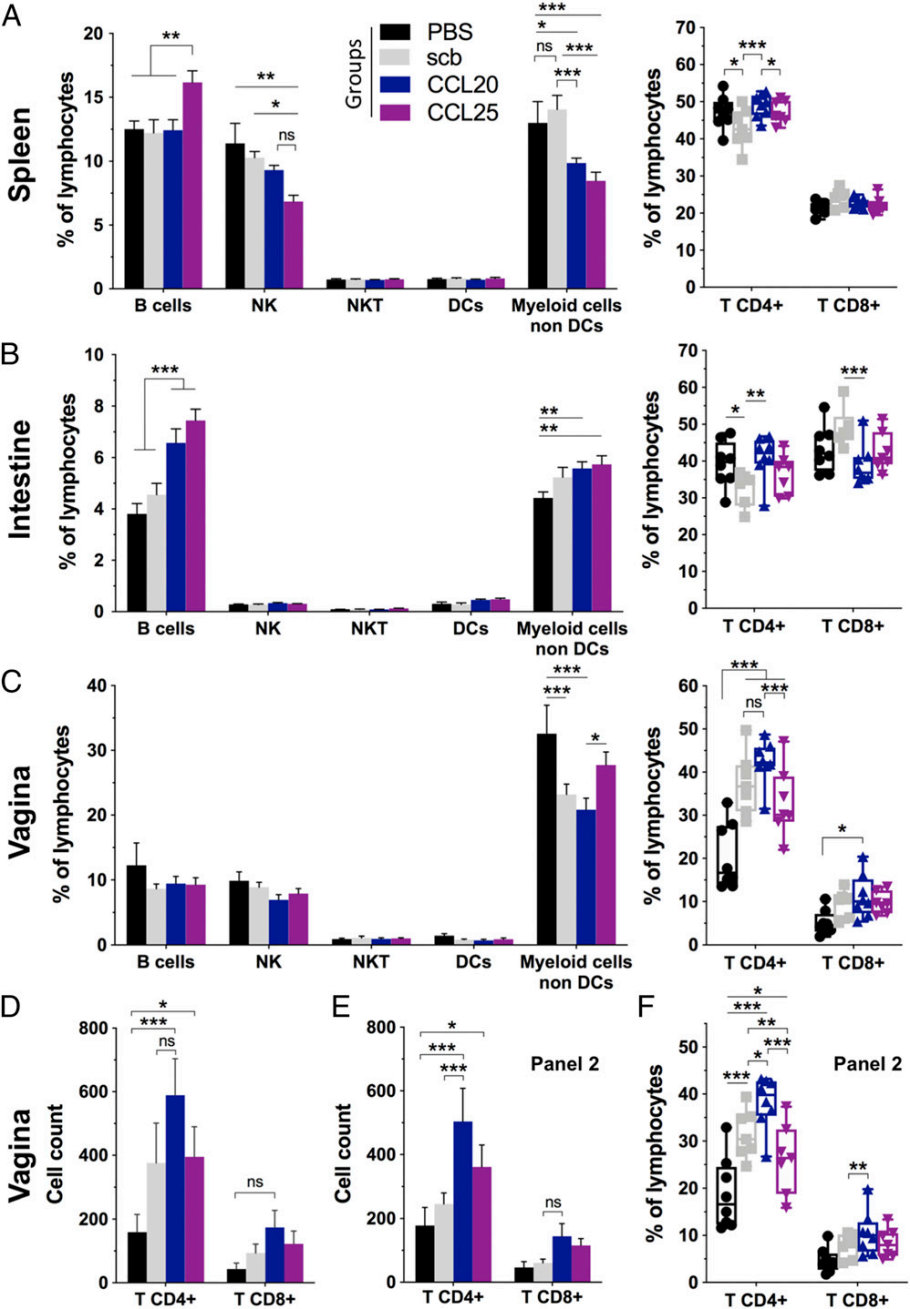
CCL20 and CCL25 adjuvanted DNA support intestinal B cell and vaginal T cell populations. (**A**) Flow cytometry analysis of lymphocytes isolated from spleens of naive (black bars), Ag–IRES–scb (gray bars), Ag–IRES–CCL20 (blue bars), and Ag–IRES–CCL25 (purple bars) and stained with mAb panel 1 (cf. Table I). The gating strategies are found in Supplemental Fig. 3A–C. The different populations are reported as percentage of lymphocytes, and the right panel depicts the proportion of CD4^+^ and CD8^+^ T cells. (**B**) and (**C**) are organized as in (A) but focusing on the small intestine and the vaginal mucosae lymphocyte populations, respectively. (**D**) and (**E**) show the total cell count of CD4^+^ and CD8^+^ T cells within the vaginal lymphocytes analyzed with panel 1 or panel 2, respectively. (**F**) Proportion of CD4^+^ and CD8^+^ T cells from the vagina as in (C) using panel 2. For panels on the left, error bars represent mean ± SEM, and for panels on the right, data points are shown as box and whiskers, min/max, with *n* = 8 animals for PBS and CCL20 groups and *n* = 7 for scb and CCL25 groups. Two-way ANOVA with Dunnett correction for multiple comparisons. *p < 0.05, **p < 0.01, ***p < 0.001. See also Supplemental Fig. 4A, 4B.

To better characterize the cellular response induced by chemokines adjuvanted DNA, four groups were selected for flow cytometry phenotypic analysis: PBS, Ag–IRES–scb, Ag–IRES–CCL20, and Ag–IRES–CCL25. We designed two flow cytometry panels to characterize the immune cell balance as well as the T cell activation and memory status (Tables I, II, Supplemental Figs. 3, 4A, 4B). When we looked at the splenocytes, a striking 4% increase in the B cell proportion was observed for the Ag–IRES–CCL25 group compared with the three other groups, whereas the proportion of CD4^+^ and CD8^+^ T cells remained the same as the PBS group (Fig. 4A). Interestingly, the CCL20 and CCL25 adjuvanted groups had lower proportion of NK and myeloid cells (non-DC) in the systemic compartment compared with PBS and Ag–IRES–scb groups. The proportion of CD4^+^ T cells was reduced in the Ag–IRES–scb group compared with PBS and Ag–IRES–CCL20 groups whereas the proportion of CD8^+^ T cells remained equivalent among the four groups. Thus, these data indicate that CCL25 and CCL20 genetic adjuvantation can modulate the systemic immune cell homeostasis.

**Table I.** Flow cytometry mAb panel 1

Flow Panel 1					
Spec.	Dye	μl	Supplier	Catalog No.; RRID	Clone
CD3	PE	1	eBiosciences	12-0031-85; AB_465498	145-2C11
CD4	Allophycocyanin	1	BioLegend	116014; AB_2563025	RM4-4
CD8	PerCP Cy5.5	1	BioLegend	100734; AB_2075238	53-6.7
CD11c	Allophycocyanin Cy7	2	BioLegend	117324; AB_830649	N418
I-Ad	FITC	2	BioLegend	115006; AB_313621	39-10-8
CD49b	PE Dazzle 594	1	BioLegend	108924; AB_2565271	DX5
CD19	PE Cy7	1	eBiosciences	25-0193-82; AB_657663	eBio1D3

Volume (μl) of Ab used per sample for panel 1 master mix.

RRID, Research Resource Identifier; Spec., specificity.

**Table II.** Flow cytometry mAb panel 2

Flow Panel 2					
Spec.	Dye	μl	Supplier	Catalog No.; RRID	Clone
CD3	PE	1	BioLegend	12-0031-85; AB_465498	145-2C11
CD4	Allophycocyanin Cy7	1	BioLegend	100414; AB_312699	GK1.5
CD8	PerCP Cy5.5	1	BioLegend	100734; AB_2075238	53-6.7
CD44	PE Cy7	1	BioLegend	103030; AB_830787	IM7
CD62L	BV421	1	BioLegend	104436; AB_2562560	MEL-14
CD103	Allophycocyanin	1.5	BioLegend	121414; AB_1227502	2E7
CD69	BV605	1	BioLegend	104530; AB_2563062	H1.2F3
CD49b	PE Dazzle 594	1	BioLegend	108924; AB_2565271	DX5

Volume (μl) of Ab used per sample for panel 2 master mix.

RRID, Research Resource Identifier; Spec., specificity.

Remarkably, lymphocytes isolated from the small intestine showed that the immune balance had been significantly altered by CCL20 and CCL25 adjuvantation with a ∼1.7 and ∼2-fold increase in the B cell proportion compared with PBS, respectively (∼1.4 and ∼1.6-fold compared with the Ag–IRES–scb control) (Fig. 4B). In addition, the Ag–IRES–CCL20 and Ag–IRES–CCL25 groups presented similar T cell percentages as the PBS group, whereas the Ag–IRES–scb group exhibited a CD8^+^ T cell enriched and CD4^+^ T cell reduced profile. No variations were observed with regard to NK, NKT and dendritic cells (DC), whereas a slight increase of non-DC myeloid cells was noted for the CCL20 and CCL25 adjuvanted groups. These observations provide evidence that CCL20 and CCL25 genetic adjuvantation induced homing of B cells to the small intestine.

### CCL20 DNA-adjuvantation induces T cell homing to the vaginal mucosa

To determine if homing to the genital tract mucosa was induced by any of the four groups selected for flow cytometry, lymphocytes isolated from the vagina were analyzed. In contrast to the intestinal lymphocytes, the four groups showed similar proportions of B cells in the vagina (Fig. 4C). NK, NKT, and DC were found in equivalent proportion across the four groups. Compared with the PBS group, the proportion of CD4^+^ T cells was increased in the vagina for Ag–IRES–scb, –CCL20, and –CCL25 groups. Thus, all DNA-primed groups induced a specific response that could home to the vaginal mucosa and/or be recruited to the vagina following the protein boost. Nevertheless, a higher proportion of CD4^+^ T and CD8^+^ T cells was observed in the CCL20 adjuvanted group compared with the Ag–IRES–scb and Ag–IRES–CCL25 groups (Fig. 4C, 4F). Moreover, these differences were not a direct consequence of variation between the relative proportion of immune cell types but rather reflected the actual number of T cells present in the genital tract as shown by the total cell count of the vaginal samples (Fig. 4D, 4E). This indicates that the CCL20 adjuvanted group had more specific CD4^+^ T and CD8^+^ T cells already residing in the vaginal mucosa, which could quickly proliferate upon local Ag exposure.

### CCL25 DNA-adjuvantation modulates the CD4^+^ T cell activation status of the systemic compartment and intestinal mucosa

**FIGURE 5.**
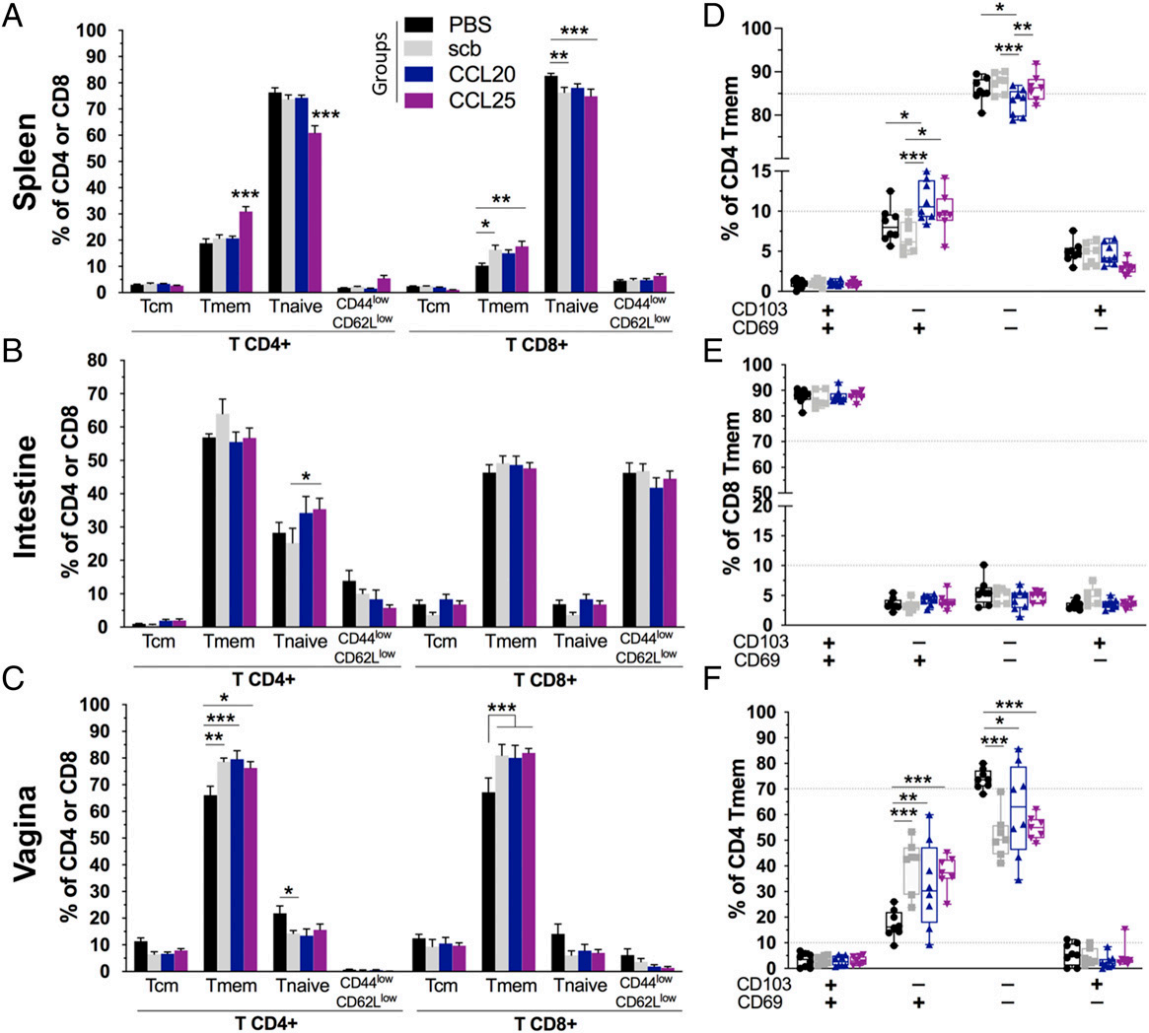
Strong memory CD4^+^ T cell recall following intravaginal Ag exposure in Ag–IRES–CCL25 immunized mice. Flow cytometry analysis of lymphocytes isolated from spleens (**A**), small intestine (**B**), and vaginal mucosa (**C**) of naive (black bars), Ag–IRES–scb (gray bars), Ag–IRES–CCL20 (blue bars), and Ag–IRES–CCL25 (purple bars) and stained with mAb panel 2 (cf. Table II). The gating strategies are found in Supplemental Figs. 3D, 4A, and 4B. The different T cell subpopulations are reported as percent of CD4^+^ or CD8^+^ T cells with naive T CD44^low^ CD62L^high^, Tmem CD44^high^ CD62L^low^, and central memory T CD44^high^ CD62L^high^. The panels on the right depict the surface expression of CD69/CD103 as percentage of CD4^+^ Tmem in the spleen (**D**) and in the vaginal mucosa (**F**) as well as in the intestinal mucosa for CD8^+^ Tmem (**E**). For panels on the left, error bars represent mean ± SEM, and for panels on the right, data points are shown as box and whiskers, min/max, with *n* = 8 animals per group and *n* = 7 for Ag–IRES–scb and Ag–IRES–CCL25. Two-way ANOVA with Dunnett correction for multiple comparisons. *p < 0.05, **p < 0.01, ***p < 0.001. See also Supplemental Fig. 4C–E.

Subsequently, we looked at the T cell subsets using CD44 and CD62L to discriminate between naive T, memory T (Tmem), and central memory T phenotypes (Fig. 5A–C, Supplemental Figs. 3B, 4A, 4B). The proportion of systemic CD4^+^ Tmem appeared greatly increased in the Ag–IRES–CCL25 group with 30.1% of the CD4^+^ T cells being CD44^high^ CD62L^low^ compared with the other groups (18.9–20.1%) (Fig. 5A). In contrast, we observed a ∼1.5-fold increase in the proportion of CD8^+^ Tmem in the spleen for the Ag–IRES–scb, –CCL20, and –CCL25 groups compared with the PBS control group. Analysis of the intestinal T cells showed that the T cell subsets balance was not affected in this compartment (Fig. 5B). In marked contrast, the vaginal CD8^+^ T and CD4^+^ T cells appeared skewed toward the Tmem phenotype, with a >10% increase for the three immunized groups compared with the PBS control mice (Fig. 5C). Therefore, these phenotypic data suggest that CCL25 DNA-adjuvantation can induce CD4^+^ T cell memory responses following DNA immunization that are efficiently recalled upon boosting at a distant site (i.e., vaginal mucosa) and that these cells were programmed in such a way that they could recirculate and/or proliferate more efficiently in the systemic compartment than the Ag–IRES–scb and –CCL20 groups.

Finally, as tissue-resident memory T cells (Trm) are key in initiating a rapid protective response against pathogens at barrier tissues (32–35), we sought to determine whether CCL20 and CCL25 genetic adjuvantation could alter Tmem residency. With this aim, we characterized the surface expression of CD69 and CD103 residency markers on CD4^+^ CD44^high^ CD62L^low^ and CD8^+^ CD44^high^ CD62L^low^ Tmem cells (Fig. 5D–F, Supplemental Figs. 3D, 4). As expected, the vast majority of Tmem from the spleen were CD103^−^ CD69^−^ conventional T effector memory cells, but interestingly, we observed a slight increase in the proportion of CD103^−^ CD69^+^ CD4^+^ Tmem for the CCL20 and CCL25 adjuvanted groups compared with the *scb* and PBS groups (Fig. 5D). This implies that these cells have been activated and therefore suggest an efficient recall of specific CD4^+^ T cells from the systemic compartment upon topical Ag immunization. In contrast, all immunized groups presented a slight reduction in CD103^−^ CD69^+^ CD8^+^ Tmem together with an increase of CD103^−^ CD69^−^ CD8^+^ conventional T effector memory cells (Supplemental Fig. 4C). In addition, the phenotyping of vaginal lymphocytes revealed that activated CD103^−^ CD69^+^ CD4^+^ Tmem were found in higher proportions in the vaginal mucosa for the Ag–IRES–scb, –CCL20, and –CCL25 groups compared with the PBS group, likely reflecting an effective recruitment and activation of CD4^+^ T cells following topical Ag exposure and local inflammation (Fig. 5F). We could also observe an increased proportion of CD103^−^ CD69^+^ CD8^+^ Tmem in the vagina for the Ag–IRES–scb and –CCL25 groups, yet to a lesser extent (Supplemental Fig. 4E). No differences were observed between the immunized groups and the PBS group for the intestinal mucosa with regard to CD103/CD69 markers (Fig. 5E, Supplemental Fig. 4D). In addition, our results also showed no variation between the four groups regarding the proportion of CD8^+^ CD44^high^ CD62L^low^ CD103^+^ CD69^+^ cells, which constitutes the majority of CD8^+^ Trm in the intestine. This may suggest that this residency phenotype was not specifically programmed by CCL20 or CCL25 adjuvantation. However, the Ag specificity of the Tmem cells was not assessed in our study and we cannot exclude that the proportion of Ag-specific cells within the Trm population may have been increased by chemokine adjuvantation.

## Discussion

Eliciting an appropriate immune response at mucosal sites is likely necessary to protect from sexually transmitted pathogens such as HIV and *Chlamydia*, respiratory pathogens like influenza and RSV, or gastrointestinal infections caused by rotavirus, clostridium, and *Salmonella*. To induce mucosal immunity, strategies involving systemic and topical immunizations with chemokine adjuvants have been tested with the aim to recruit and program immune cells (8, 9, 11, 18, 28). In this study, we developed a DNA platform which allows bicistronic expression of the Ag of interest and a chosen chemokine/cytokine using an EMCV IRES. Using this platform, we evaluated the potential of four chemokines (CCL20, CCL25, CCL27, CCL28) and one cytokine (TNF-α) to program T and B cells to home to the female genital tract and/or to the intestinal mucosa upon systemic DNA immunization and a subsequent vaginal protein boost in mice.

We showed that CCL20 genetic adjuvantation induced a strong systemic IgG response that was boosted after topical Ag exposure in the genital tract. Moreover, the substantial serum IgG titer enhancement observed for the CCL25 adjuvanted group suggested that following DNA immunizations, a proportion of the reactive Ag-specific immune cells homed to the vaginal mucosa, where they could be activated upon topical Ag exposure. In contrast, Ag–IRES–CCL27, –CCL28, and –TNF-α groups only showed a 1.4–2.5-fold increase of the systemic Ag-specific IgG following vaginal boost. This suggests that these chemokines were not as effective in programming B cells to home to the female genital tract and/or induce an adequate memory phenotype for recall. Moreover, although it has been shown that TNF-α can enhance the HIV-1 Env gp120 serum IgG response when delivered as a genetic fusion gp120–TNF-α expression plasmid (28), in this study it did not enhance the systemic gp140-specific IgG response compared with the Ag–IRES–scb control.

Although eliciting neutralizing Abs, which do not require Fc function to mediate protection, are a primary goal for HIV-1 vaccine, the RV144 HIV-1 clinical trial showed that the weak protection observed was linked to nonneutralizing Env-specific IgG3 that could mediate Ab-dependent cellular cytotoxicity (36). Therefore, being able to modulate the quality of the response by inducing class-switching from IgG1 to IgG3 may be key to developing protective vaccines. In mice, the class-switch from IgG1 to IgG2 (similar to IgG3 in human) is supported by Th1 type responses and therefore we looked at the IgG subtype specificity. We observed that following DNA immunizations the Ag–IRES–scb group presented a mixed profile with Th1/Th2 balanced or Th1 skewed responses, which contrast with other reports where Env immunogens derived from other HIV-1 strains and delivered by DNA induced a Th2 skew (37–39). Although immunization with HIV-1 Gag along with CCL20 DNA plasmids has previously been shown to induce a Th1 response (40), our results showed that CCL20 genetic adjuvantation induced a marked Th2 skew following DNA immunization. Although two further studies investigated the use of CCL20 genetic adjuvantation of a HCV core plasmid DNA and of a foot-and-mouth disease vaccine, the IgG1 and IgG2a responses were assessed solely after Ag plus Montanide ISA 206 adjuvant boost, showing a Th1 profile (18, 41). In our study, despite the use of MPLA (known to induce strong Th1 responses) the Ag–IRES–CCL20 group maintained its Th2 skewed profile following the Ag plus adjuvant topical boost. Whether the differences in T helper responses are due to the studied Ag, the quantity of DNA used (20× less in our study compared with Ref. 18) or the adjuvant used will require further investigations. In addition, following DNA immunization Ag–IRES–CCL25, –CCL27, –CCL28, and –TNF-α induced a mixture of Th1 skewed, Th2 skewed and balanced Th1/Th2 responses which persisted post protein boost. As other studies using these chemokines/cytokine for DNA vaccination purposes did not measure the Ag-specific IgG1 and IgG2a, assessing their role in the induction of a Th1 or Th2 response remains to be clarified (8, 9, 26, 42).

In this study, we showed that CCL20 and CCL25 adjuvanted groups presented higher Ag-specific and total IgA levels in the intestinal mucosa compared with the other groups. Moreover, flow cytometry data analyzing the intestinal lymphocytes confirmed that Ag–IRES–CCL20 and –CCL25 groups had a higher proportion of B cells than the PBS and the Ag–IRES–scb groups. The CCL25 adjuvanted group also presented an increase of the systemic B cell pool, whereas Ag–IRES–CCL20 systemic pool remained similar to the PBS and the Ag–IRES–scb groups, suggesting a more specific programming of B cells using CCL20 genetic adjuvantation. Altogether, these results indicate that CCL20 and CCL25 induced homing of B cells to the intestinal mucosa and that a proportion of these cells were programmed to secrete IgA. However, although Ag-specific B cells homed to the intestinal mucosa, we demonstrated that non–Ag-specific bystander B cells also homed to the intestinal mucosa, highlighting the importance of measuring and reporting both the Ag-specific and total Ab levels.

Looking at the cellular response, the CCL28 adjuvanted group showed the highest proportion of specific IFN-γ secreting T cells in the vagina, whereas its systemic response was similar to Ag–IRES–scb, –CCL20, –CCL27, and –TNF-α groups. In contrast, the CCL25 adjuvanted group presented the lowest specific IFN-γ response for both the systemic and vaginal compartments, whereas the proportion of CD4^+^ T and CD8^+^ T cells remained similar to the Ag–IRES–scb group. This suggests that CCL25 genetic adjuvantation limited the extent of the T cell response following DNA immunizations, whereas a previous study showed that CCL25 enhanced the IFN-γ T cell specific response when codelivered with Gag or HA Ags (9). The Ag–IRES–CCL20 group had similar systemic and vaginal IFN-γ responses compared with the Ag–IRES–scb group but presented a significantly higher proportion of CD4^+^ T cells in these compartments. Moreover, the increased CD4^+^ T cell population in the vagina induced by CCL20 genetic adjuvantation appeared to be linked to a higher number of T cells and was therefore not an artificial increase related to other cell type populations decreasing. Taken together, these observations indicate that CCL20 genetic adjuvantation efficiently programmed specific T cells to home to the vaginal mucosa but, similarly to B cells, non–Ag-specific bystander T cells may be part of the total T cell pool programmed to home to the vaginal mucosa. Finally, the activation and memory status of T cells within the intestinal lymphocyte pool (PP, IEL, and LPL) and the vaginal lymphocyte pool were characterized. Of note, the animals were not perfused with PBS before tissue collection, and therefore circulating cells from the systemic compartment may be found in the T cell populations. No marked differences were observed between Ag–IRES–scb, –CCL20, and –CCL25 with the exception of Ag–IRES–CCL25 showing a significant increase in the effector memory CD4^+^ T cell population in the spleen. This suggests that the CD4^+^ T cell memory response shaped by CCL25 adjuvantation could efficiently be recalled following Ag exposure at a distant site. When we looked at residency markers, both CCL20 and CCL25 chemokines did not appear to impact the proportion of Trm in the intestinal and vaginal tissues. Although the cell frequencies varied depending on the chemokine used, we did not assess the Ag-specific proportion of B and T cells. Therefore, we cannot exclude that cells residing within nonlymphoid tissues such as Trm may contain a higher proportion of Ag-specific cells for CCL20 and CCL25 groups.

In conclusion, our study compared a panel of chemokines/cytokine for genetic adjuvant use and demonstrated in a mouse model that although these genetic adjuvants can induce homing of Ag-specific B and T cells to the vaginal and intestinal mucosae, non–Ag-specific bystander B cells and possibly bystander T cells are also programmed to home to these sites. Thus, assessing the specificity of this bystander cellular and humoral responses in these compartments following chemokine/cytokine genetic adjuvantation will be critical in preventing possible auto-reactive responses triggered by these adjuvants.

## Supplementary Material

Data Supplement

## References

[1] Bomsel M. Alfsen A. 2003 Entry of viruses through the epithelial barrier: pathogenic trickery. Nat. Rev. Mol. Cell Biol. 4: 57–68. 12511869 10.1038/nrm1005 PMC7097689

[2] Real F. Sennepin A. Ganor Y. Schmitt A. Bomsel M. 2018 Live imaging of HIV-1 transfer across T cell virological synapse to epithelial cells that promotes stromal macrophage infection. Cell Rep. 23: 1794–1805. 29742434 10.1016/j.celrep.2018.04.028

[3] Gao Y. McKay P. F. Mann J. F. S. 2018 Advances in HIV-1 vaccine development. Viruses 10: E167. 29614779 10.3390/v10040167 PMC5923461

[4] Li Q. Zeng M. Duan L. Voss J. E. Smith A. J. Pambuccian S. Shang L. Wietgrefe S. Southern P. J. Reilly C. S. 2014 Live simian immunodeficiency virus vaccine correlate of protection: local antibody production and concentration on the path of virus entry. J. Immunol. 193: 3113–3125. 25135832 10.4049/jimmunol.1400820 PMC4157131

[5] Moldt B. Le K. M. Carnathan D. G. Whitney J. B. Schultz N. Lewis M. G. Borducchi E. N. Smith K. M. Mackel J. J. Sweat S. L. 2016 Neutralizing antibody affords comparable protection against vaginal and rectal simian/human immunodeficiency virus challenge in macaques. AIDS 30: 1543–1551. 27243773 10.1097/QAD.0000000000001102 PMC4915739

[6] Kiniry B. E. Li S. Ganesh A. Hunt P. W. Somsouk M. Skinner P. J. Deeks S. G. Shacklett B. L. 2018 Detection of HIV-1-specific gastrointestinal tissue resident CD8^+^ T-cells in chronic infection. Mucosal Immunol. 11: 909–920. 29139476 10.1038/mi.2017.96 PMC5953759

[7] Su F. Patel G. B. Hu S. Chen W. 2016 Induction of mucosal immunity through systemic immunization: phantom or reality? Hum. Vaccin. Immunother. 12: 1070–1079. 26752023 10.1080/21645515.2015.1114195 PMC4962944

[8] Kutzler M. A. Kraynyak K. A. Nagle S. J. Parkinson R. M. Zharikova D. Chattergoon M. Maguire H. Muthumani K. Ugen K. Weiner D. B. 2010 Plasmids encoding the mucosal chemokines CCL27 and CCL28 are effective adjuvants in eliciting antigen-specific immunity in vivo. Gene Ther. 17: 72–82. 19847203 10.1038/gt.2009.112 PMC10751736

[9] Kathuria N. Kraynyak K. A. Carnathan D. Betts M. Weiner D. B. Kutzler M. A. 2012 Generation of antigen-specific immunity following systemic immunization with DNA vaccine encoding CCL25 chemokine immunoadjuvant. Hum. Vaccin. Immunother. 8: 1607–1619. 23151454 10.4161/hv.22574 PMC3601135

[10] Shin H. Iwasaki A. 2012 A vaccine strategy that protects against genital herpes by establishing local memory T cells. Nature 491: 463–467. 23075848 10.1038/nature11522 PMC3499630

[11] Sun X. Zhang H. Xu S. Shi L. Dong J. Gao D. Chen Y. Feng H. 2017 Membrane-anchored CCL20 augments HIV Env-specific mucosal immune responses. Virol. J. 14: 163. 28830557 10.1186/s12985-017-0831-4 PMC5568278

[12] Sanders R. W. van Gils M. J. Derking R. Sok D. Ketas T. J. Burger J. A. Ozorowski G. Cupo A. Simonich C. Goo L. 2015 HIV-1 VACCINES. HIV-1 neutralizing antibodies induced by native-like envelope trimers. Science 349: aac4223. 26089353 10.1126/science.aac4223 PMC4498988

[13] Pugach P. Ozorowski G. Cupo A. Ringe R. Yasmeen A. de Val N. Derking R. Kim H. J. Korzun J. Golabek M. 2015 A native-like SOSIP.664 trimer based on an HIV-1 subtype B env gene. J. Virol. 89: 3380–3395. 25589637 10.1128/JVI.03473-14 PMC4337520

[14] Sarkar A. Bale S. Behrens A. J. Kumar S. Sharma S. K. de Val N. Pallesen J. Irimia A. Diwanji D. C. Stanfield R. L. 2018 Structure of a cleavage-independent HIV Env recapitulates the glycoprotein architecture of the native cleaved trimer. Nat. Commun. 9: 1956. 29769533 10.1038/s41467-018-04272-y PMC5955915

[15] Aldon Y. McKay P. F. Allen J. Ozorowski G. Felfodine Levai R. Tolazzi M. Rogers P. He L. de Val N. Fabian K. 2018 Rational design of DNA-expressed stabilized native-like HIV-1 envelope trimers. Cell Rep 24: 3324–3338.e5. 30232012 10.1016/j.celrep.2018.08.051 PMC6167709

[16] Kong L. He L. de Val N. Vora N. Morris C. D. Azadnia P. Sok D. Zhou B. Burton D. R. Ward A. B. 2016 Uncleaved prefusion-optimized gp140 trimers derived from analysis of HIV-1 envelope metastability. Nat. Commun. 7: 12040. 27349805 10.1038/ncomms12040 PMC4931249

[17] Sharma S. K. de Val N. Bale S. Guenaga J. Tran K. Feng Y. Dubrovskaya V. Ward A. B. Wyatt R. T. 2015 Cleavage-independent HIV-1 Env trimers engineered as soluble native spike mimetics for vaccine design. Cell Rep. 11: 539–550. 25892233 10.1016/j.celrep.2015.03.047 PMC4637274

[18] Hartoonian C. Sepehrizadeh Z. Mahdavi M. Arashkia A. Jang Y. S. Ebtekar M. Yazdi M. T. Negahdari B. Nikoo A. Azadmanesh K. 2014 Modulation of hepatitis C virus core DNA vaccine immune responses by co-immunization with CC-chemokine ligand 20 (CCL20) gene as immunoadjuvant. Mol. Biol. Rep. 41: 5943–5952. 24972567 10.1007/s11033-014-3470-5

[19] Flingai S. Czerwonko M. Goodman J. Kudchodkar S. B. Muthumani K. Weiner D. B. 2013 Synthetic DNA vaccines: improved vaccine potency by electroporation and co-delivered genetic adjuvants. Front. Immunol. 4: 354. 24204366 10.3389/fimmu.2013.00354 PMC3816528

[20] Schutyser E. Struyf S. Van Damme J. 2003 The CC chemokine CCL20 and its receptor CCR6. Cytokine Growth Factor Rev. 14: 409–426. 12948524 10.1016/s1359-6101(03)00049-2

[21] Papadakis K. A. Prehn J. Nelson V. Cheng L. Binder S. W. Ponath P. D. Andrew D. P. Targan S. R. 2000 The role of thymus-expressed chemokine and its receptor CCR9 on lymphocytes in the regional specialization of the mucosal immune system. J. Immunol. 165: 5069–5076. 11046037 10.4049/jimmunol.165.9.5069

[22] Hieshima K. Kawasaki Y. Hanamoto H. Nakayama T. Nagakubo D. Kanamaru A. Yoshie O. 2004 CC chemokine ligands 25 and 28 play essential roles in intestinal extravasation of IgA antibody-secreting cells. J. Immunol. 173: 3668–3675. 15356112 10.4049/jimmunol.173.6.3668

[23] Reiss Y. Proudfoot A. E. Power C. A. Campbell J. J. Butcher E. C. 2001 CC chemokine receptor (CCR)4 and the CCR10 ligand cutaneous T cell-attracting chemokine (CTACK) in lymphocyte trafficking to inflamed skin. J. Exp. Med. 194: 1541–1547. 11714760 10.1084/jem.194.10.1541 PMC2193675

[24] Lazarus N. H. Kunkel E. J. Johnston B. Wilson E. Youngman K. R. Butcher E. C. 2003 A common mucosal chemokine (mucosae-associated epithelial chemokine/CCL28) selectively attracts IgA plasmablasts. J. Immunol. 170: 3799–3805. 12646646 10.4049/jimmunol.170.7.3799

[25] Kunkel E. J. Butcher E. C. 2002 Chemokines and the tissue-specific migration of lymphocytes. Immunity 16: 1–4. 11825560 10.1016/s1074-7613(01)00261-8

[26] Kutzler M. A. Wise M. C. Hutnick N. A. Moldoveanu Z. Hunter M. Reuter M. Yuan S. Yan J. Ginsberg A. Sylvester A. 2016 Chemokine-adjuvanted electroporated DNA vaccine induces substantial protection from simian immunodeficiency virus vaginal challenge. Mucosal Immunol. 9: 13–23. 25943275 10.1038/mi.2015.31 PMC4636490

[27] Su B. Wang J. Wang X. Jin H. Zhao G. Ding Z. Kang Y. Wang B. 2008 The effects of IL-6 and TNF-alpha as molecular adjuvants on immune responses to FMDV and maturation of dendritic cells by DNA vaccination. Vaccine 26: 5111–5122. 18462845 10.1016/j.vaccine.2008.03.089

[28] Nimal S. Heath A. W. Thomas M. S. 2006 Enhancement of immune responses to an HIV gp120 DNA vaccine by fusion to TNF alpha cDNA. Vaccine 24: 3298–3308. 16464521 10.1016/j.vaccine.2006.01.020

[29] Mann J. F. Tregoning J. S. Aldon Y. Shattock R. J. McKay P. F. 2016 CD71 targeting boosts immunogenicity of sublingually delivered influenza haemagglutinin antigen and protects against viral challenge in mice. J. Control Release 232: 75–82. 27094605 10.1016/j.jconrel.2016.04.022

[30] Sheridan B. S. Lefrançois L. 2012 Isolation of mouse lymphocytes from small intestine tissues. Curr. Protoc. Immunol. Chapter 3: Unit3.19. 10.1002/0471142735.im0319s99 PMC8128104 23129154

[31] Peng S. L. Szabo S. J. Glimcher L. H. 2002 T-bet regulates IgG class switching and pathogenic autoantibody production. Proc. Natl. Acad. Sci. USA 99: 5545–5550. 11960012 10.1073/pnas.082114899 PMC122806

[32] Srivastava R. Hernández-Ruiz M. Khan A. A. Fouladi M. A. Kim G. J. Ly V. T. Yamada T. Lam C. Sarain S. A. B. Boldbaatar U. 2018 CXCL17 chemokine-dependent mobilization of CXCR8^+^CD8^+^ effector memory and tissue-resident memory T cells in the vaginal mucosa is associated with protection against genital herpes. J. Immunol. 200: 2915–2926. 29549178 10.4049/jimmunol.1701474 PMC5893430

[33] Sheridan B. S. Pham Q. M. Lee Y. T. Cauley L. S. Puddington L. Lefrançois L. 2014 Oral infection drives a distinct population of intestinal resident memory CD8(+) T cells with enhanced protective function. Immunity 40: 747–757. 24792910 10.1016/j.immuni.2014.03.007 PMC4045016

[34] Ariotti S. Hogenbirk M. A. Dijkgraaf F. E. Visser L. L. Hoekstra M. E. Song J. Y. Jacobs H. Haanen J. B. Schumacher T. N. 2014 T cell memory. Skin-resident memory CD8^+^ T cells trigger a state of tissue-wide pathogen alert. Science 346: 101–105. 25278612 10.1126/science.1254803

[35] Gebhardt T. Wakim L. M. Eidsmo L. Reading P. C. Heath W. R. Carbone F. R. 2009 Memory T cells in nonlymphoid tissue that provide enhanced local immunity during infection with herpes simplex virus. Nat. Immunol. 10: 524–530. 19305395 10.1038/ni.1718

[36] Corey L. Gilbert P. B. Tomaras G. D. Haynes B. F. Pantaleo G. Fauci A. S. 2015 Immune correlates of vaccine protection against HIV-1 acquisition. Sci. Transl. Med. 7: 310rv7. 10.1126/scitranslmed.aac7732 PMC4751141 26491081

[37] Ye L. Wen Z. Dong K. Pan L. Bu Z. Compans R. W. Zhang H. Yang C. 2010 Immunization with a mixture of HIV Env DNA and VLP vaccines augments induction of CD8 T cell responses. J. Biomed. Biotechnol. 2010: 497219. 20508832 10.1155/2010/497219 PMC2876254

[38] Storcksdieck genannt Bonsmann M. Niezold T. Temchura V. Pissani F. Ehrhardt K. Brown E. P. Osei-Owusu N. Y. Hannaman D. Hengel H. Ackerman M. E. 2015 Enhancing the quality of antibodies to HIV-1 envelope by GagPol-specific Th cells. J. Immunol. 195: 4861–4872. 26466954 10.4049/jimmunol.1501377

[39] Daly L. M. Johnson P. A. Donnelly G. Nicolson C. Robertson J. Mills K. H. 2005 Innate IL-10 promotes the induction of Th2 responses with plasmid DNA expressing HIV gp120. Vaccine 23: 963–974. 15603899 10.1016/j.vaccine.2004.03.072

[40] Song R. Liu S. Leong K. W. 2007 Effects of MIP-1 alpha, MIP-3 alpha, and MIP-3 beta on the induction of HIV Gag-specific immune response with DNA vaccines. Mol. Ther. 15: 1007–1015. 10.1038/mt.sj.6300129 PMC2365720 17356539

[41] Jayeshbhai C. Hajam I. A. Verma A. K. Bhanuprakash V. Kondabattula G. Kishore S. 2018 Chemokine CCL20 plasmid improves protective efficacy of the Montanide ISA™ 206 adjuvanted foot-and-mouth disease vaccine in mice model. Vaccine 36: 5318–5324. 30054161 10.1016/j.vaccine.2018.07.003

[42] Kraynyak K. A. Kutzler M. A. Cisper N. J. Khan A. S. Draghia-Akli R. Sardesal N. Y. Lewis M. G. Yan J. Weiner D. B. 2010 Systemic immunization with CCL27/CTACK modulates immune responses at mucosal sites in mice and macaques. Vaccine 28: 1942–1951. 20188250 10.1016/j.vaccine.2009.10.095 PMC4396814

