# Supplementary material for: Chemokine-Adjuvanted Plasmid DNA Induces Homing of Antigen-Specific and Non–Antigen-Specific B and T Cells to the Intestinal and Genital Mucosae
Source: J Immunol. 2020 Jan 8;204(4):903–13. doi: 10.4049/jimmunol.1901184 (PMC6994839; doi:10.4049/jimmunol.1901184)
Supplement: Data Supplement [file JI_1901184.zip › JI_1901184_Supplemental_Figures_1.pdf]

Supplementary Figures

A

GCTAGCGCCGCCACCATGGACAGAGCCAAACTCCTCCTTCTCCTTTTGCTCCTGCTGCTGCCTCAGGCCCAGGCTGTGGGAAACCTGTGGGTCACCGTGTACTACGGCGTGCCCGTGTGGAA
GGGCGCCACCACCACACTGTTCTGTGCCTCTGACGCCAAGGCCTACGACACCGAGGTGCACAACGTGTGGGCCACCCACGCTTGTGTGCCTGCCGATCCCAACCCCCAGGAAATGGTGCTGG
AAAACGTGACCGAGAACTTCAACATGTGGAAGAACGAGATGGTCAACCAGATGCAGGAAGATGTGATCAGCCTGTGGGACCAGAGCCTGAAGCCCTGCGTGAAGCTGACCCCTCTGTGCGTG
ACCCTGGAATGCAGAAACGTGTCCAGCAACAGCAACGACACCTACCACGAGACATACCACGAGTCCATGAAGGAAATGAAGAACTGCAGCTTCAACGCCACCACAGTCGTGCGGGACAGAAA
GCAGACCGTGTACGCCCTGTTCTACAGACTGGACATCGTGCCCCTGACCAAGAAGAACTACAGCGAGAACAGCAGCGAGTACTACCGGCTGATCAACTGCAACACCAGCGCCATCACCCAGG
CCTGCCCCAAAGTGACCTTCGACCCCATCCCCATCCACTACTGTACCCCTGCCGGCTACGCCATCCTGAAGTGCAACGACAAGATTTTCAACGGCACCGGCCCCTGCCACAATGTGTCCACC
GTGCAGTGTACCCACGGCATCAAGCCCGTGGTGTCCACCCAGCTGCTGCTGAACGGATCTCTGGCCGAGGGCGAGATCATCATCAGAAGCGAGAACCTGACCAACAACGTGAAAACCATCAT
CGTGCATCTGAACCAGAGCGTGGAAATCGTGTGCACCAGACCCGGCAACAACACCAGAAAGAGCATCAGAATCGGCCCTGGCCAGACCTTCTACGCCACCGGCGATATTATCGGCGACATCA
GACAGGCCCACTGCAACATCTCCGAGGACAAGTGGAACGAGACACTGCAGAGAGTGTCCAAGAAGCTGGCCGAGCACTTCCAGAACAAGACCATCAAGTTCGCCAGCTCCTCTGGCGGCGAC
CTGGAAGTGACCACCCACAGCTTTAACTGCAGAGGCGAGTTCTTCTACTGTAACACCTCCGGCCTGTTCAACGGCGCCTACACCCCTAACGGCACCAAGAGCAACTCCAGCAGCATCATCAC
CATCCCTTGCAGGATCAAGCAGATCATCAATATGTGGCAGGAAGTGGGCAGGGCTATGTACGCCCCTCCCATCAAGGGCAACATCACATGCAAGTCCAACATCACCGGACTGCTGCTCGTGC
GCGACGGCGGAACAGAGCCCAACGACACAGAGACATTCAGACCTGGCGGCGGAGACATGAGAAACAACTGGCGGAGCGAGCTGTACAAGTACAAGGTGGTGGAAATCAAGCCCCTGGGCGTG
GCCCCTACCACCACCAAGAGAAGAGTGGTGGAACGCGAGAAGGCCGCCGTGGGAATCGGCGCTGTGTTCCTGGGATTTCTGGGAGTGGCCGGCAGCACAATGGGCGCTGCCAGCATCACACT
GACCGTGCAGGCTAGACAGCTGCTGAGCGGCATCGTGCAGCAGCAGAGTAACCTGCTGAGAGCCATCGAGGCACAGCAGCATCTGCTGCAGCTGACCGTGTGGGGAATCAAGCAGCTGCAGA
CCAGAGTGCTGGCCATCGAGAGATACCTGAAGGACCAGCAGCTGCTGGGCATCTGGGGCTGTAGCGGCAAGCTGATCTGTACCACCGCCGTGCCCTGGAACAGCTCCTGGTCCAACAAGTCC
CAGAAAGAAATCTGGGACAACATGACCTGGATGCAGTGGGACAAAGAGATCAGCAACTACACCAACACAGTGTACAGGCTGCTGGAAGAGAGCCAGAACCAGCAGGAAAGAAACGAGAAGGA
CCTGCTGGCCCTGGACTCC**TAATAA**GGATCC**TGACTAACTAG**AATTAATTCGCTGTCTGCGAGGGCCAGCTGTTGGGGTGAGTACTCCCTCTCAAAAGCGGGCATGACTTCTGCGCTAAGAT
TGTCAGTTTCCAAAAACGAGGAGGATTTGATATTCACCTGGCCCGCGGTGATGCCTTTGAGGGTGGCCGCGTCCATCTGGTCAGAAAAGACAATCTTTTTGTTGTCAAGCTTGAGGTGTGGC
AGGCTTGAGATCTGGCCATACACTTGAGTGACAATGACATCCACTTTGCCTTTCTCTCCACAGGTGTCCACTCCCAGGTCCAACTGCAGGTCGAGCATGCATCTAGGGCGGCCAATTCCGCC
CCTCTCCCCCCCCCCCCTCTCCCTCCCCCCCCCCTAACGTTACTGGCCGAAGCCGCTTGGAATAAGGCCGGTGTGCGTTTGTCTATATGTTATTTTCCACCATATTGCCGTCTTTTGGCAAT
GTGAGGGCCCGGAAACCTGGCCCTGTCTTCTTGACGAGCATTCCTAGGGGTCTTTCCCCTCTCGCCAAAGGAATGCAAGGTCTGTTGAATGTCGTGAAGGAAGCAGTTCCTCTGGAAGCTTC
TTGAAGACAAACAACGTCTGTAGCGACCCTTTGCAGGCAGCGGAACCCCCCACCTGGCGACAGGTGCCTCTGCGGCCAAAAGCCACGTGTATAAGATACACCTGCAAAGGCGGCACAACCCC
AGTGCCACGTTGTGAGTTGGATAGTTGTGGAAAGAGTCAAATGGCTCTCCTCAAGCGTATTCAACAAGGGGCTGAAGGATGCCCAGAAGGTACCCCATTGTATGGGATCTGATCTGGGGCCT
CGGTGCACATGCTTTACATGTGTTTAGTCGAGGTTAAAAAAACGTCTAGGCCCCCCGAACCACGGGGACGTGGTTTTCCTTTGAAAAACACGATGATAAGCTTGCCACAACCCGGAATTC**AT**
GGCCTGCGGTGGCAAGCGTCTGCTCTTCCTTGCTTTGGCATGGGTACTGCTGGCTCACCTCTGCAGCCAGGCAGAAGCAGCAAGCAACTACGACTGTTGCCTCTCGTACATACAGACGCCTC
TTCCTTCCAGAGCTATTGTGGGTTTCACAAGACAGATGGCCGATGAAGCTTGTGACATTAATGCTATCATCTTTCACACGAAGAAAAGAAAATCTGTGTGCGCTGATCCAAAGCAGAACTGG
GTGAAAAGGGCTGTGAACCTCCTCAGCCTAAGAGTCAAGAAGATG**TAATAA**CTCGAG

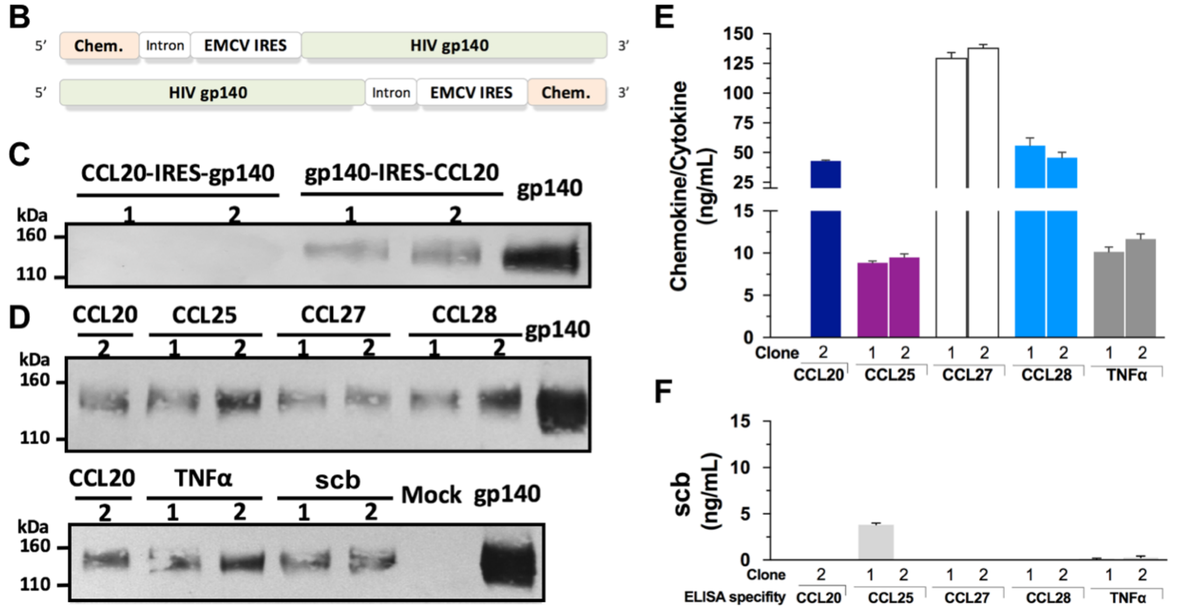

Supp. Figure 1. IRES bi-cistronic expression cassette for HIV-1 Env gp140 and chemokine. (**A**) DNA nucleotide sequence of the HIV-1 Env CN54 gp140-IRES-CCL20 cloned into pcDNA3.1. Underlined: nucleotide excluded from codon optimization. Red: Kozak sequence. **TAATAA**: two stop codons at the end of CN54 gp140 and CCL20 open reading frame. Restriction site for cloning of other antigens or chemokines: GCTAGC, NheI-HF; GGATCC, BamHI-HF; GAATTC, EcoRI-HF; CTCGAG, XhoI-HF. Elements for bi-cistronic expression: **TGACTAACTAG**, extra stop codons in the 3 reading frames; Green, spacer in 5' chimeric intron; GTGAG---ACAG, chimeric intron; Orange, spacer 3' chimeric intron; Blue, encephalomyocarditis Virus (EMCV) Internal Ribosome Entry Site (IRES); Purple, spacer 3' of the IRES before ATG start of second gene. (**B**) Schematic of the bi-cistronic expression insert with two configurations: (top) CN54gp140 downstream of and under the control of encephalomyocarditis virus (EMCV) internal ribosome entry site (IRES) or (bottom) upstream of the EMCV IRES. (**C**) Two clones of pcDNA3.1 expressing CCL20-IRES-gp140 and two clones expressing gp140-IRES-CCL20 were probed by Western blotting for gp140 expression in the supernatant of transfected HEK293T.17 cells. A protein control (gp140) well is included. (**D**) Two clones of each gp140-IRES-chemokine/cytokine (CCL25, CCL27, CCL28, TNFα and scrambled CCL20 (scb)) expressing vector were tested by Western blot for gp140 expression in the supernatant of transfected HEK293T.17 cells. (**E**) and (**F**) Chemokine/cytokine expression was evaluated by capture ELISA using the supernatant sample from (**C**). The chemokine on the x-axis indicate both the specificity of the ELISA and the chemokine/cytokine cloned into the expressing vector in (**E**) while in (**F**) the x-axis indicates the specificity of the ELISA against which the two gp140-IRES-sc expressing clones have been tested. Chem. = chemokine/cytokine; Mock = empty pcDNA3 expression vector control; scb, *scrambled CCL20.*

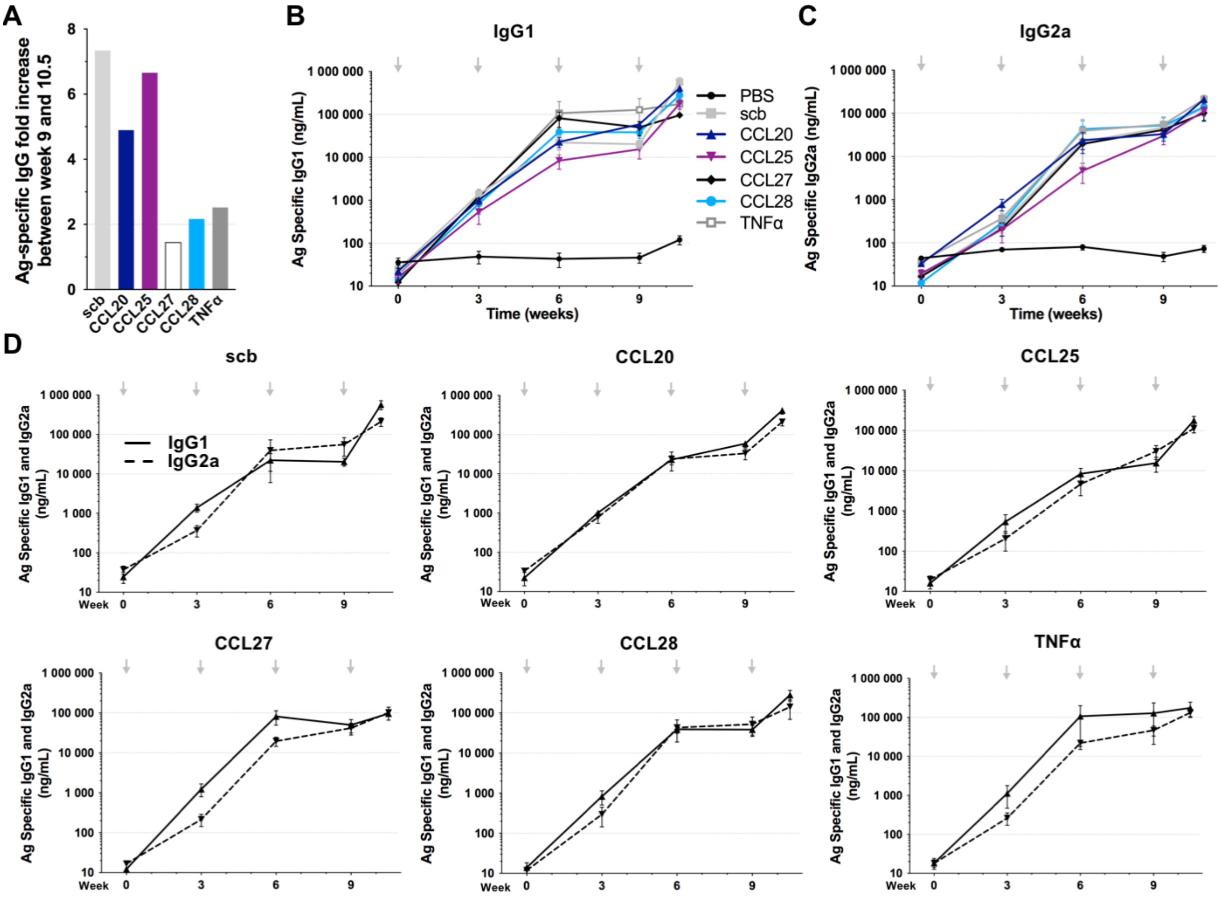

Supp. Figure 2. Serum specific IgG and subtype IgG1 and IgG2a. Related to **Figure 1. (A)** CN54gp140 specific IgG mean value fold increase between week 9 and 10.5. **(B)** and **(C)** Serum IgG1 and IgG2a antigen-specific ELISA results, respectively. Error bars represent mean ± SEM. **(D)** Serum IgG1 (plain line) and IgG2a (dashed line) titers for each individual chemokine/cytokine tested. Arrows indicate immunizations. scb, *scrambled CCL20*.

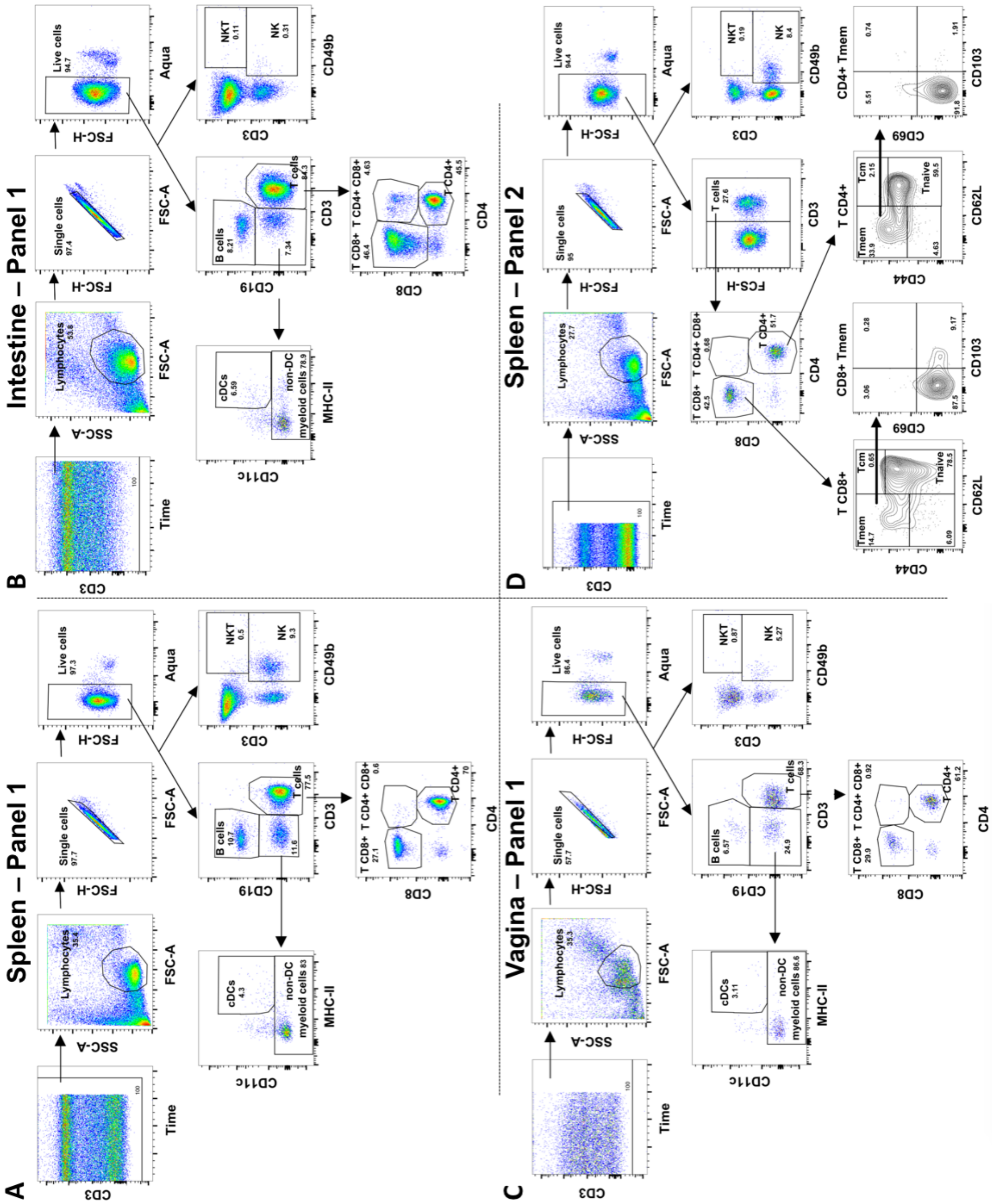

Supp. Figure 3. Flow cytometry gating strategies for Balb/c mice lymphocytes using panel 1 and panel 2. Related to **Figure 4** and **5.** The gating strategies are depicted for panel 1 **(A)** spleen, **(B)** intestine, **(C)** vagina and for panel 2 **(D)** spleen. The name of each population gated is indicated together with its relative percentage with regard to the parent population. For the spleen, an animal from the naïve PBS control group is shown here; for the intestine, an animal from the CN54gp140-IRES-CCL25 group is shown; for the vagina, an animal from the CN54gp140-IRES-CCL20 group is shown.

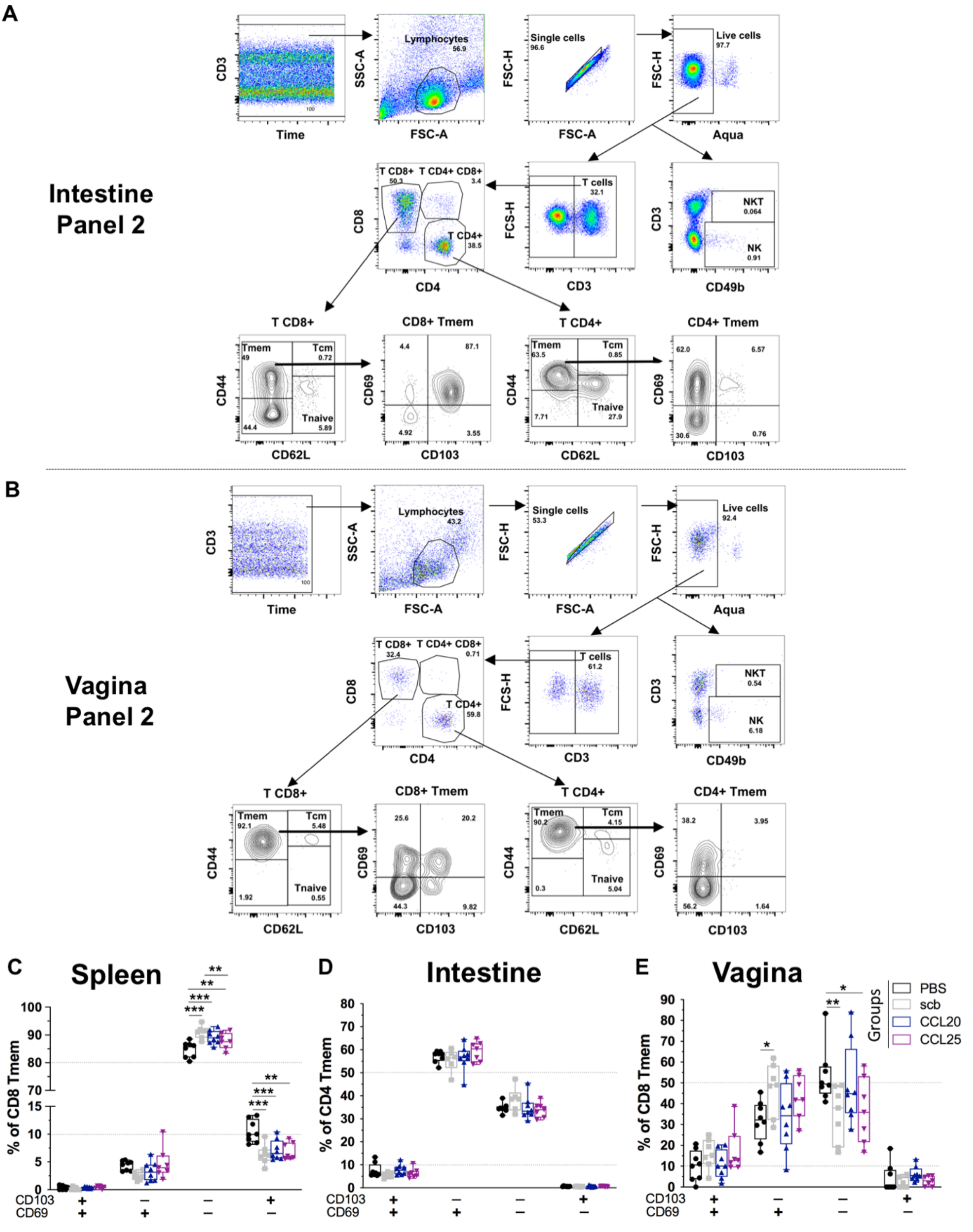

Supp. Figure 4. Gating strategies of panel 2 and expression of CD69 and CD103 on Tmem. Related to **Figure 4** and **5.** The gating strategies for panel 2 **(A)** intestine and **(B)** vagina are depicted. **(D)** spleen. Flow cytometry analysis of Tmem from the spleen **(C)**, intestine **(D)** and vaginal mucosae **(E)** of naïve (black), Ag-IRES-scb (grey), Ag-IRES-CCL20 (blue) and Ag-IRES-CCL25 (purple) and stained with panel 2 (cf. **Table II**). The panels **(C-D)** depict the surface expression of CD69/CD103 as percent of CD4+ Tmem or CD8+ Tmem. Box and whiskers, min./max., with n = 8 animals per groups and n = 7 for Ag-IRES-scb and Ag-IRES-CCL25. Two-way ANOVA with Dunnett's correction for multiple comparisons, *p* values: *<0.05, **<0.01, ***<0.001. scb = *scrambled CCL20*.
